# Single‐cell transcriptomics reveals IRF7 regulation of the tumor microenvironment in isocitrate dehydrogenase wild‐type glioma

**DOI:** 10.1002/mco2.754

**Published:** 2024-11-03

**Authors:** Jinwei Li, Shengrong Long, Zhang Yang, Wei Wei, Shuangqi Yu, Quan Liu, Xuhui Hui, Xiang Li, Yinyan Wang

**Affiliations:** ^1^ Department of Neurosurgery Beijing Tiantan Hospital Capital Medical University Beijing China; ^2^ Beijing Neurosurgical Institute Capital Medical University Beijing China; ^3^ Department of Neurosurgery West China Hospital Sichuan University Chengdu China; ^4^ Department of Neurosurgery Zhongnan Hospital of Wuhan University Wuhan China; ^5^ Brain Research Center Zhongnan Hospital of Wuhan University Wuhan China; ^6^ Department of Vascular Surgery Fuwai Yunnan Cardiovascular Hospital Affiliated Cardiovascular Hospital of Kunming Medical University Kunming China; ^7^ Department of Neurosurgery The Fourth Affiliated Hospital of Guangxi Medical University Liuzhou China

**Keywords:** gene transcription regulation, glioma, interferon regulatory factor 7, isocitrate dehydrogenase 1, multi‐omics

## Abstract

Mutations in isocitrate dehydrogenase (IDH) are important markers of glioma prognosis. However, few studies have examined the gene expression regulatory network (GRN) in IDH‐mutant and wild‐type gliomas. In this study, single‐cell RNA sequencing and spatial transcriptome sequencing were used to analyze the GRN of cell subsets in patients with IDH‐mutant and wild‐type gliomas. Through gene transcriptional regulation analysis, we identified the M4 module, whose transcription factor activity is highly expressed in IDH wild‐type gliomas compared to IDH‐mutants. Enrichment analysis revealed that these genes were predominantly expressed in microglia and macrophages, with significant enrichment in interferon‐related signaling pathways. Interferon regulatory factor 7 (IRF7), a transcription factor within this pathway, showed the highest percentage of enrichment and was primarily localized in the core region of wild‐type IDH tumors. A machine‐learning prognostic model identified novel subgroups within the wild‐type IDH population. Additionally, IRF7 was shown to promote the proliferation and migration of T98G and U251 cells in vitro, and its knockdown affected glioma cell proliferation in vivo. This study systematically established the regulatory mechanism of IDH transcriptional activity in gliomas at the single‐cell level and drew a corresponding cell map. The study presents a transcriptional regulatory activity map for IDH wild‐type gliomas, involving single‐cell RNA sequencing and spatial transcriptomics to identify gene regulatory networks, machine learning models for IDH subtyping, and experimental validation, highlighting the role of IRF7 in glioma progression.

## Graphical abstract

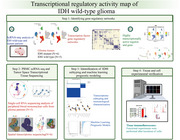

## 1 INTRODUCTION

Gliomas are the most common primary intracranial tumors, accounting for over 80% of malignant brain tumors.^1^ Glioma treatment primarily relies on surgical resection, followed by postoperative radiotherapy and chemotherapy. However, the therapeutic outcomes in most patients remain unsatisfactory. In 2008, the discovery of isocitrate dehydrogenase (IDH) mutations in a subgroup of diffuse infiltrating gliomas, ushering in a new era of glioma molecular classification. In 2016, the World Health Organization (WHO) incorporated the mutation status of IDH into the diagnosis of diffuse infiltrating gliomas as part of the classification of central nervous system tumors.^2^ The recent 2021 WHO classification defined a unique tumor family based on these molecular alterations, which encompasses gliomas ranging from low to high grade and is distinct from IDH wild‐type glioblastomas at the molecular level.^3^ IDH mutations are recognized as early driver mutations and form the molecular pathological basis of the modern glioma classification.^4^ Although significant progress has been made in the molecular classification of gliomas through the identification of IDH mutations, the factors driving glioma progression remain unclear.

Gliomas are stratified based on the IDH mutation status. The WHO clinical guidelines explicitly state that IDH has significant implications for the diagnosis, personalized treatment, and clinical prognostic assessment of brain gliomas. Currently, wild‐type IDH is considered the most important factor for the definitive diagnosis of glioblastoma.^3^ The median survival time of patients with glioblastoma is less than 15 months, and the 5‐year overall survival rate is only 6.8%.^5^ Patients with wild‐type IDH have a poorer prognosis than those with IDH mutation.^3^ Single‐cell studies have shown key differences in the genetic heterogeneity of malignant cell states between IDH‐mutant gliomas and wild‐type IDH tumors, with the latter exhibiting a higher degree of cellular plasticity.^6^ Therefore, exploring the tumor microenvironment (TME) characteristics of wild‐type IDH gliomas is important.

The study of tumor heterogeneity is complex because of cellular plasticity, which allows cancer cells to transition reversibly between different cellular states in response to genetic, microenvironmental, and therapeutic stimuli.^7^, ^8^ Epigenetic regulators of cellular plasticity and environmental stress responses have been elucidated using single‐cell transcriptome sequencing (scRNA‐seq) of IDH‐mutant gliomas.^9^ The transcriptional state of a cell is derived from a potential gene regulatory network (GRN) that includes transcription factors (TFs), cofactors, and their downstream target genes.^10^ Co‐expression modules between TFs and potential target genes can be efficiently identified using single‐cell regulatory network inference and clustering (SCENIC). Previous studies have also demonstrated the dynamic cellular states in wild‐type IDH gliomas.^11^ IDH‐mutant gliomas exhibit limited plasticity along the hierarchical differentiation axis.^12^, ^13^ Interplay between cancer cell genotypes, expression programs associated with cellular phenotypes, and the influence of the TME control tumor adaptability, evolution, and resistance to therapy.^14^ The GRN analyzed by scRNA‐seq provides an exciting opportunity to identify transcriptional states and transitions at high resolution.^10^ Therefore, there is an urgent need for simultaneous GRN reconstruction and cell‐state characterization of gliomas with different IDH statuses.

In this study, we performed a comprehensive GRN analysis using scRNA‐seq on TME cells from five IDH‐mutants and six wild‐type gliomas. Highly active TFs were specifically overexpressed in wild‐type IDH microglial cells. Most TFs were enriched in the interferon (IFN)‐related signaling pathway, and *IRF7* was one of the most important genes in the IFN‐signaling pathway. Therefore, we knocked down *IRF7* in various cell lines to verify its biological function and simultaneously performed tissue immunofluorescence localization. Spatial localization and metabolism of the IRF7 regulon and IRF7 were verified in situ using six cases of spatial transcriptome sequencing: IDH wild‐type glioblastoma, glioblastoma cell tumors with secondary surgery, and paraneoplastic tissues. Clustering and typing of IDH wild‐type gliomas was undertaken using the IRF7 regulon. In summary, our study identifying the GRN in patients with wild‐type IDH gliomas provided exciting results and showed that the IRF7‐associated IFN signaling pathway may influence patient prognosis.

## 2 RESULTS

### 2.1 IDH‐mutant and wild‐type glioma TME composition

**FIGURE 1:**
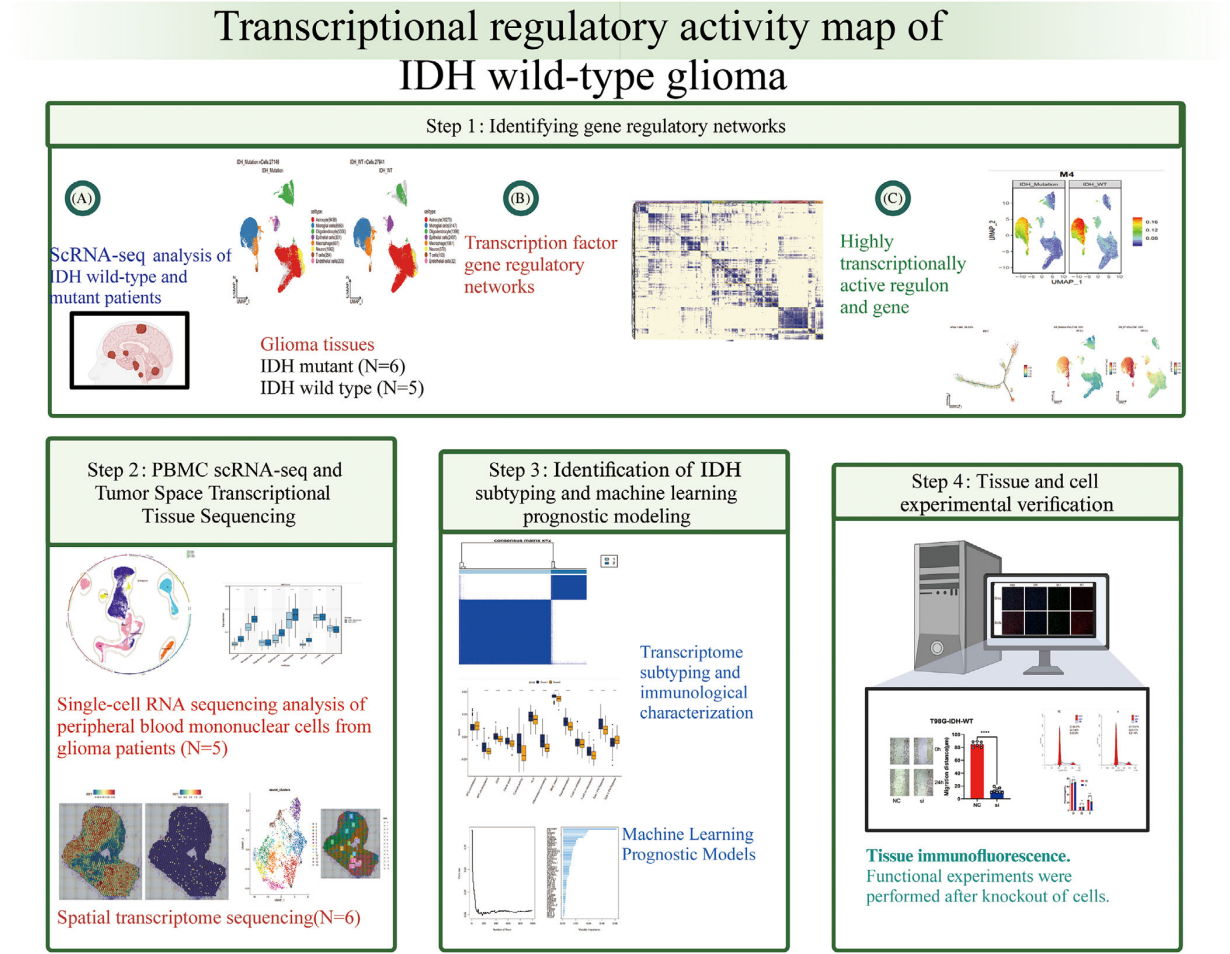
Research design roadmap.

We analyzed gene transcriptional regulation in patients with IDH‐mutant and wild‐type IDH gliomas through a four‐step strategy (Figure 1). In the first step, regulatory networks were analyzed for changes in transcriptional activity in IDH‐mutant and wild‐type gliomas. In the second step, the expression of key genes was verified by single‐cell sequencing of the glioma blood mononuclear cells, and by spatial transcriptome analysis of key gene localization in different glioma tissues. Third, wild‐type IDH subtypes were identified, and prognostic models were constructed. In the fourth step, experiments were performed to verify the key genes.

**FIGURE 2:**
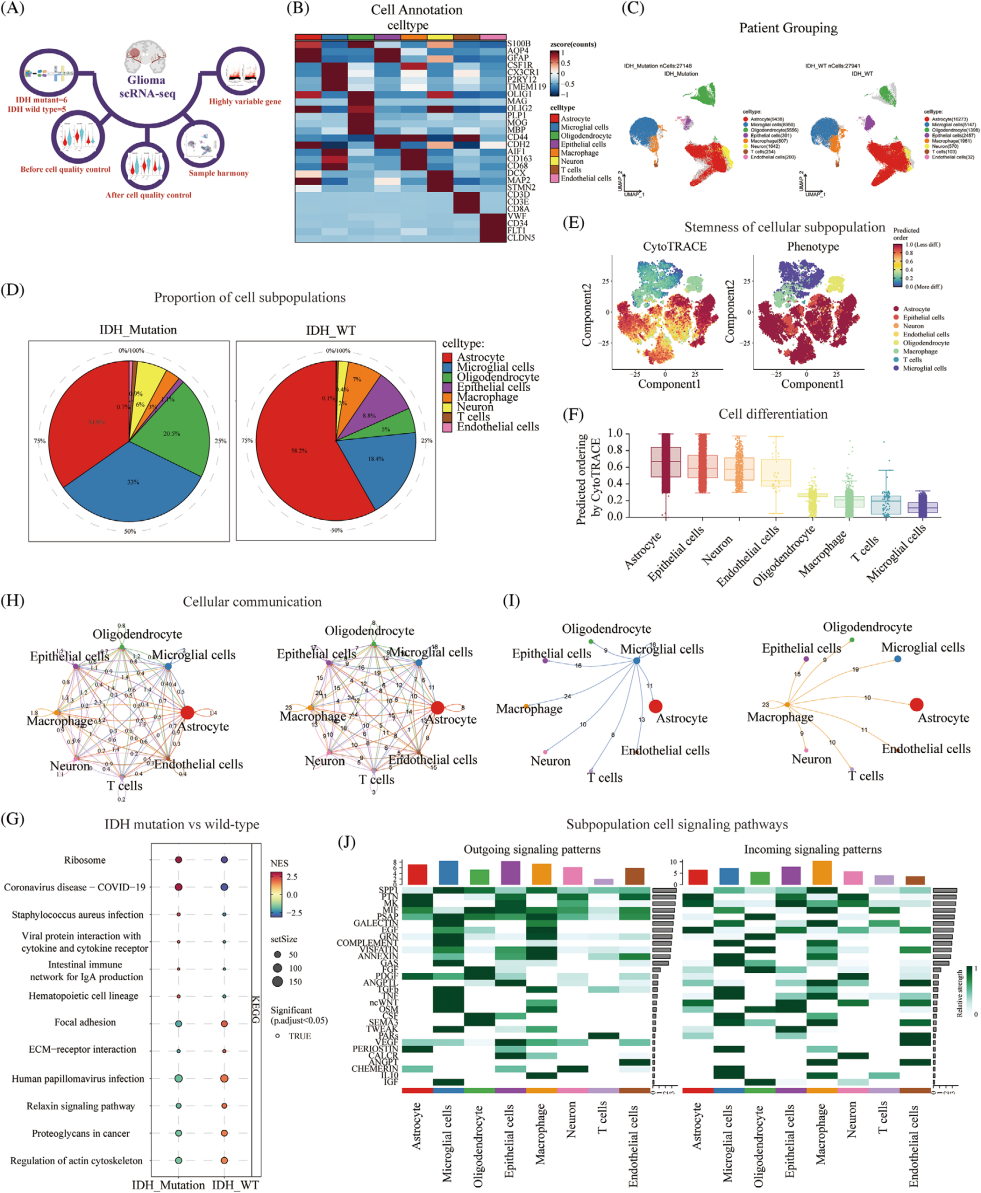
The tumor microenvironment of isocitrate dehydrogenase (IDH) wild‐type and mutant glioma tumor tissues. (A) Quality control process. (B) Heatmap showed cell subsets annotated marker. (C) Uniform Manifold Approximation and Projection (UMAP) showed the distribution of IDH‐mutant and wild‐type cell subpopulations. (D) Cell ratio histogram showed the proportion of cell subpopulations. (E) UMAP showed the differentiation of cell subpopulations in terms of stemness. (F) Bar graph showed the stemness score of cell subpopulations. (G) Scatter plot showed the signaling pathways enriched by IDH‐mutant and wild‐type gene set enrichment analysis (GSEA). (H) The circular network diagram showed the weight and number of interactions between cell subsets. (I) The circular network map showed the number of microglia and macrophages communicating with other cells. (J) The heatmap showed the input and output signal pathways of cell subsets.

In the first step, we analyzed single‐cell sequencing data to investigate the composition of the immune microenvironment in wild‐type and mutant IDH tumors. The quality control process used during the early stages of data processing is shown in Figure 2A. A heatmap was generated to display the expression of marker genes for cell annotation (Figure 2B). The Uniform Manifold Approximation and Projection (UMAP) plot showed 27,148 cells in the IDH‐mutant group and 27,941 cells in the IDH wild‐type group, including astrocytes, microglia, oligodendrocytes, epithelial cells, macrophages, neurons, T cells, and endothelial cells (Figure 2C). The plot of cell proportions revealed that astrocytes accounted for 58.2% in patients with wild‐type IDH, followed by microglial cells (18.4%), oligodendrocytes (5%), and macrophages (7%). In patients with IDH mutations, astrocytes accounted for 34.8% of the cells, followed by microglia (33%), oligodendrocytes (20.5%), and macrophages (3%) (Figure 2D). CytoTRACE was used to evaluate the differentiation potential of the different single‐cell subsets. The UMAP and box charts showed that the score of the differentiation potential of microglia was the lowest (Figure 2E,F). Gene set enrichment analysis (GSEA) showed that focal adhesions, extracellular matrix (ECM)‒receptor interactions, proteoglycans in cancer, and actin cytoskeleton regulation were enriched in patients with wild‐type IDH (Figure 2G). CellChat inferred and calculated the cell‒cell communication network of single‐cell subsets of gliomas. The circular network diagram showed that microglia and macrophages had a high weight and the high number of ligand receptors in the subsets of cells (Figure 2H,I). Input signaling pathways with the highest percentage of microglia included PSAP, GALECTIN, GRN, GAS, transforming growth factor‐beta (TGF‐β), tumor necrosis factor (TNF), ncWNT, and OSM. The output signaling pathways with the highest percentage of macrophages were SPP1, MK, MIF, GALECTIN, VISFATIN, and GAS (Figure 2J).

### 2.2 GRNs in IDH‐mutant and wild‐type gliomas

**FIGURE 3:**
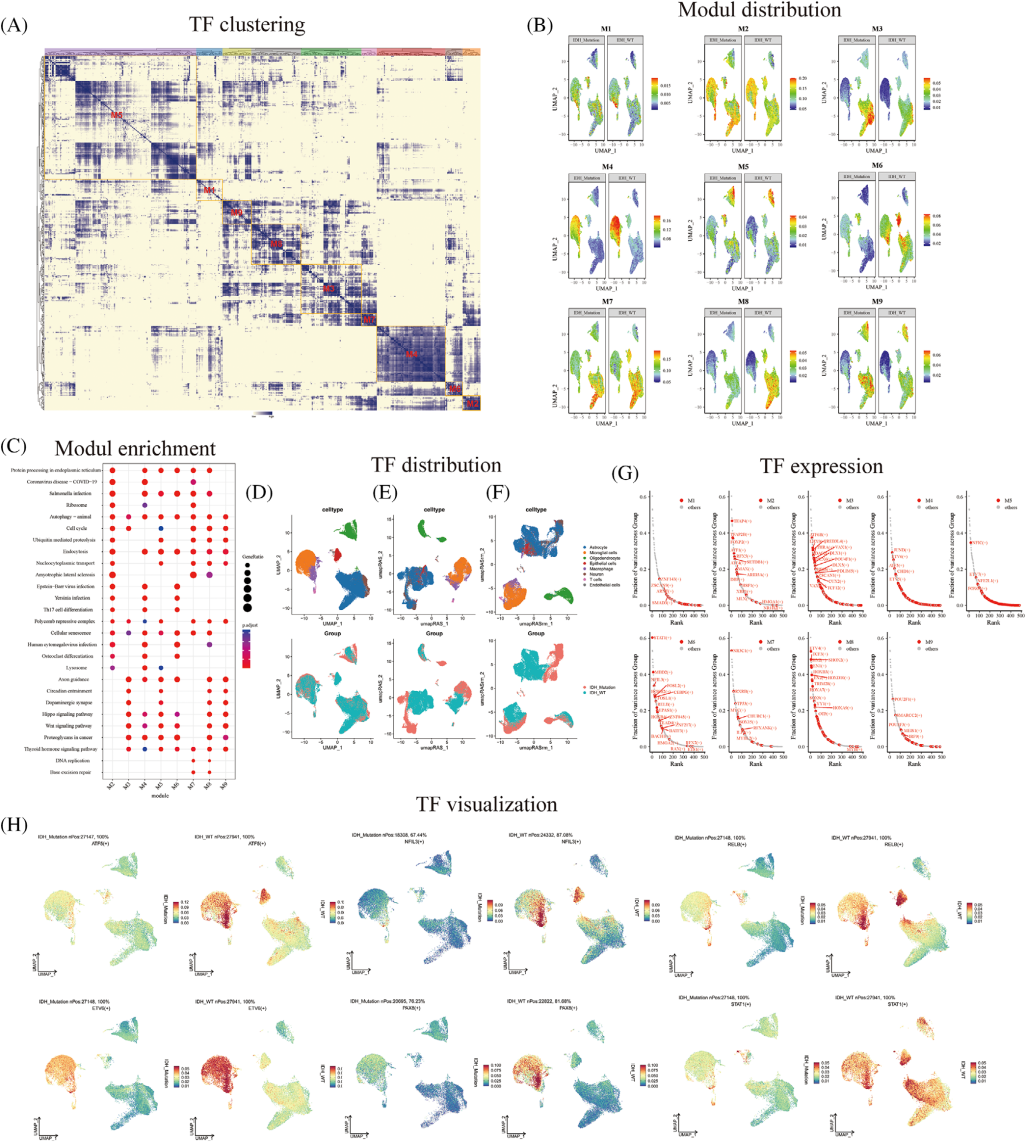
Isocitrate dehydrogenase (IDH) wild‐type and IDH‐mutant glioma transcription factor regulatory network. (A) Heatmap showed transcription factor clustering to identify highly transcriptionally active modules. (B) Uniform Manifold Approximation and Projection (UMAP) showed the distribution of transcription factor modules in IDH‐mutant and wild‐type. (C) The scatter plot showed the signal pathway of Modul enrichment. (D) UMAP showed the distribution of glioma gene subsets and groups. (E) UMAP showed the distribution of cell subsets and groups of transcription factor activity in glioma cells. (F) After disturbing four Modul group transcription factors, the subsets and groups of glioma cells with transcription factor activity were distributed. (G) The expression of transcription factors in different modules. (H) UMAP showed highly expressed transcription factors in the module.

We analyzed the differences in transcriptional regulation between IDH‐mutant and wild‐type gliomas. A cluster heatmap was generated to display a total of nine modules (Figure 3A). To identify the cell types that had the greatest impact on transcriptional activity in IDH wild‐type tumors, the UMAP plot showed that the transcriptional factor activity in module M4 differed the most between the IDH wild‐type and mutant tumors (Figure 3B). Gene enrichment analysis was performed for each transcriptional factor module. Module M4 was mainly enriched in the Wnt, Hippo, Th17 cells, cellular senescence, and cell cycle pathways (Figure 3C). The UMAP plot displays the gene expression distribution of cell subtypes in both wild‐type and mutant IDH tumors (Figure 3D). Additionally, the UMAP plot showed the distribution of RAS in the two groups of patients, revealing inconsistent RAS distribution between the two groups (Figure 3E). After perturbing the M4 module, the UMAP plot displayed the distribution of genes and changes in TFs within cell subtypes (Figure 3F). Furthermore, we identified TFs with high activity in different modules (Figure 3G). Among them, the M4 module showed high expression of TFs, such as EVT6, NFIL3, ATF5, PAX8, RELB, and STAT1 (Figure 3H).

### 2.3 Identification of TFs and signaling pathways that may regulate IDH mutation and wild‐type

GSEA enrichment analysis using wild‐type and mutant IDH microglia revealed that the activated signaling pathways were angiogenesis, oxidative phosphorylation, IFN‐α response, IFN‐γ response, and hypoxia (Figure S1A). Our analysis revealed that IRF7, IRF1, SPI1, ETS2, PRDM1, and FLI1 mediated the IFN‐α response signaling pathway (Figure S1B), while IRF7, SPI1, IRF1, ETS2, PRDM1, and FLI1 mediated the IFN‐γ response signaling pathway (Figure S1C). IRF7, SPI1, and IRF1 regulated the transcriptional regulatory network of IFN‐α response and IFN‐γ response pathway (Figure S1D). Compared to the IDH‐mutant cell subsets, IRF7 was highly expressed in IDH wild‐type microglial gliomas (Figure S1E). There was no significant difference between SPI1 and IRF1 in gliomas with different IDH statuses (Figure S1F,G). Most IRF1 regulons were found to be expressed in microglia (Figure S1H). GSEA was performed to visualize whether the IFN signaling pathway was upregulated in the IDH wild‐type (Figure S1I,J).

**FIGURE 4:**
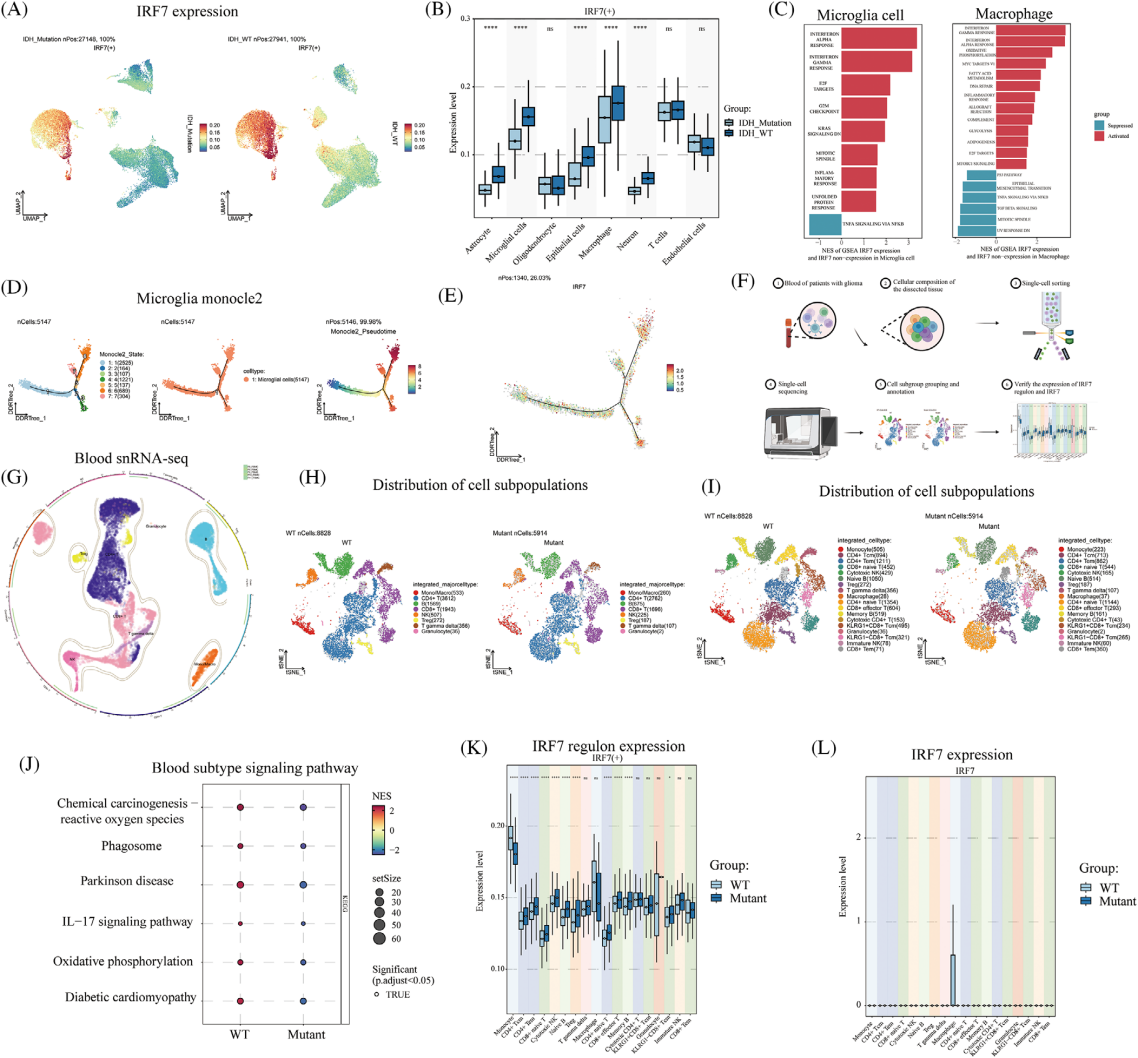
Single‐cell sequencing explored interferon regulatory factor 7 (IRF7) and IRF7 regulon expression in glioma tumors and blood. (A) Uniform Manifold Approximation and Projection (UMAP) demonstrates the distribution of IRF7 expression in isocitrate dehydrogenase (IDH) wild‐type and IDH‐mutant tumor tissues. (B) Box plots show the cellular subpopulation expression of IRF7 in IDH wild‐type and IDH‐mutant tumor tissues. (C) Enrichment analysis showed differential gene signal pathways between IRF7+ microglia and IRF7‒ microglia, and differential gene signal pathways between IRF7+ macrophages and IRF7‒ macrophages. (D) The monocle2 showed the pseudo‐sequential distribution of microglia subsets. (E) Pseudo‐sequential analysis showed the differentiation of IRF7 in microglia. (F) Procedure of single‐cell sequencing analysis of glioma IDH‐mutant and wild‐type blood. (G) The circle diagram showed the distribution of cell subsets. (H and I) UMAP showed the distribution of cell subsets in the blood microenvironment of IDH‐mutant and IDH wild‐type. (J) Scatter plot showed IDH mutation and wild‐type blood enrichment signal pathway. (K and L) The box diagram showed the expression of IRF7 regulon and IRF7 in cell subsets. ^*^ *p* < 0.05, ^**^ *p* < 0.01, ^***^ *p* < 0.001.

UMAP visualization showed that IRF7 was highly expressed in the microglia and macrophages of wild‐type IDH (Figure 4A). Compared to the IDH‐mutant cells, IRF7 was highly expressed in IDH wild‐type astrocytes, microglia, epithelial cells, macrophages, and neurons (*p* *< *0.05) (Figure 4B). GSEA was used to compare the activated and inhibited cellular pathways in IRF7+ cells and IRF7‒ microglial cells. The activated signaling pathways were IFN‐α response, IFN‐γ response, E2F targets, G2m checkpoint, KRAS signaling DN, inflammatory response, and unfolded protein response. The inhibited signaling pathway was TNF‐α signaling via nuclear factor‐kappa B (NF‐κB) (Figure 4C). GSEA was also used to compare the activated and inhibited cellular pathways in IRF7+ and IRF7‒ macrophages. The activated signaling pathways were IFN‐γ response, IFN‐α response, oxidative phosphorylation, MYC targets V1, and fatty acid metabolism. The signaling pathways inhibited were the P53 pathway, epithelial‒mesenchymal transition, TNFA signaling via NF‐κB, and TGF‐β signaling. Furthermore, we analyzed the cell trajectories of wild‐type IDH microglia. Microglioma cell cluster 6 was observed at the end of differentiation (Figure 4D). With cell differentiation and developmental trajectories, IRF7 was primarily focused on developmental differentiation endpoints (Figure 4E).

### 2.4 Single‐cell sequencing to analyze IRF7 expression in blood monocytes

We analyzed the single‐nucleus RNA sequencing (snRNA‐seq) of blood from patients with IDH‐mutant and wild‐type tumors (Figure 4F). The major subpopulations after cell annotation were monocytes/macrophages, CD4+ T cells, B cells, CD8+ T cells, NK cells, Tregs, T gamma delta cells, and granulocytes (Figure 4G,H). Sub‐population annotation classification was also performed (Figure 4I). Furthermore, we performed IDH mutation and wild‐type enrichment analyses using blood snRNA‐seq. The signaling pathways upregulated in wild‐type IDH were chemical carcinogenesis, reactive oxygen species, the interleukin (IL)‐17 signaling pathway, and oxidative phosphorylation (Figure 4J). In IDH wild‐type and mutant tumors, the expression of the IRF7 regulon was found to differ in monocytes, CD4+ Tcm, CD4+ Tem, CD8+ naïve, T cytotoxic, NK naïve, B, Treg, CD4+ naïve T, CD8+ effector T, memory B, and KLRG1‐CD8+ Tcm cells (*p* < 0.05) (Table S1 and Figure 4K). Additionally, IRF7 expression was observed in the macrophages of IDH wild‐type tumors (Figure 4L).

### 2.5 Analysis of the key IFN gene IRF7 in IDH wild‐type and mutants

**FIGURE 5:**
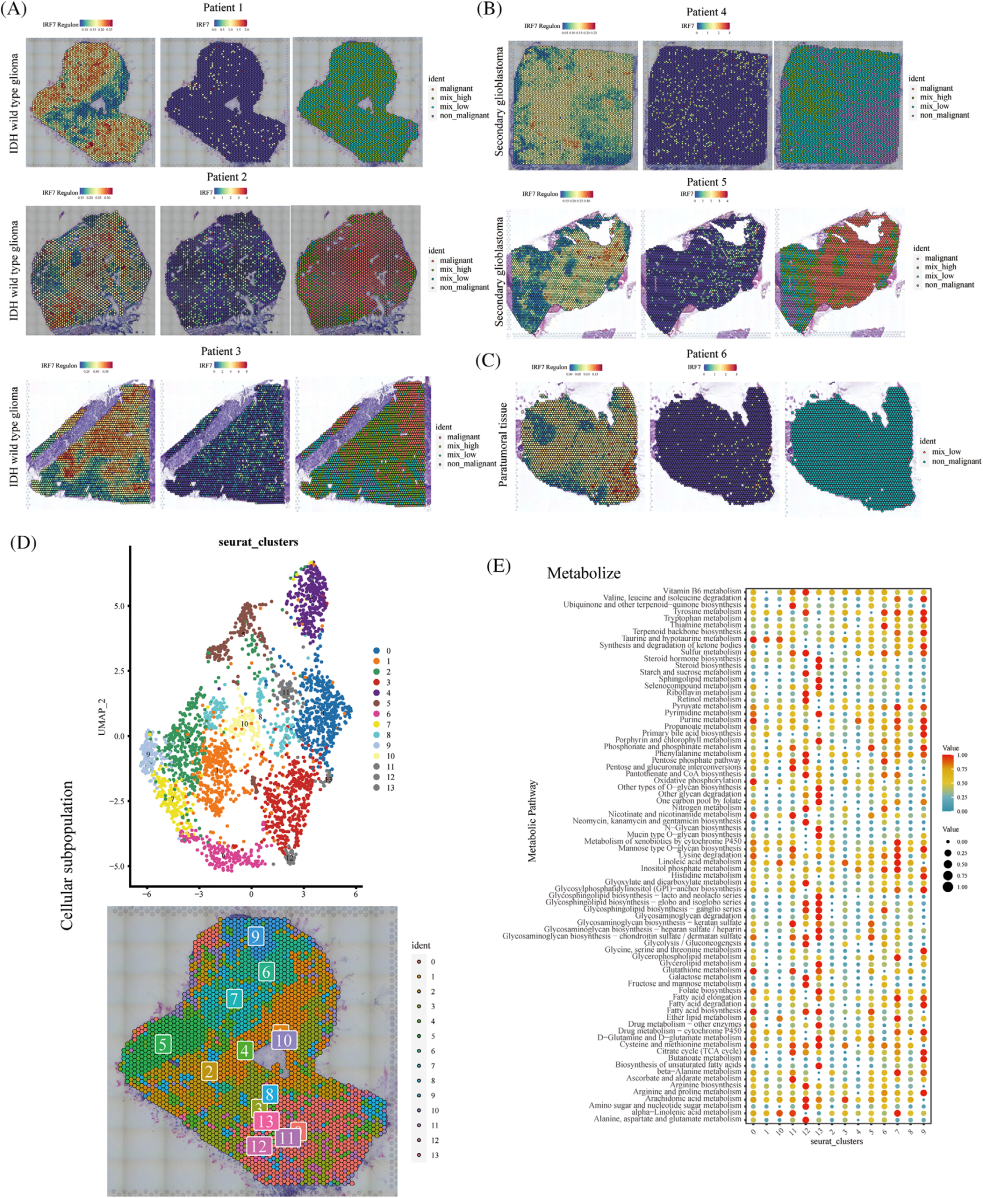
Single‐cell spatial transcriptome sequencing analysis of the spatial localization of interferon regulatory factor 7 (IRF7) regulon and IRF7. (A‒C) Spatial localization maps showing the spatial localization of key genes and malignant cell tumor content in isocitrate dehydrogenase (IDH) wild‐type gliomas, secondary glioblastomas, and paraneoplastic tissues. (D) Uniform Manifold Approximation and Projection (UMAP) and spatial localization maps showed the distribution of the IDH wild‐type subpopulation. (E) Scatterplot demonstrating metabolic signaling in IDH wild‐type subset pathways.

Subsequently, spatial transcriptome localization and expression analyses were performed. Moreover, the regions were labeled according to the degree of malignancy as malignant (malignant), mixed high (mix_high), mixed low (mix_low), and non‐malignant (non‐malignant). In wild‐type IDH gliomas, the IRF7 regulon gene was highly expressed in the center of the tumor (Figure 5A). In secondary glioblastomas, the IRF7 regulon expression was less distributed (Figure 5B). In glioblastoma tumor tissues, IRF7 regulon expression was located at the tumor margin (Figure 5C). Therefore, IRF7 regulon expression may influence tumor malignant cell content. We also performed UMAP visualization of cellular compartmentalization. In the tumor region, the metabolism‐related signaling pathway cells were predominantly IRF7 regulon high‐expression locations (Figure 5D). Finally, in IDH wild‐type glioblastomas, metabolic signaling pathways of cell subpopulations with high IRF7 regulon expression, including tyrosine metabolism, purine metabolism, pentose phosphate pathway, cysteine, and methionine metabolism, were observed (Figure 5E).

We investigated the role of the IFN‐related gene IRF7 in IDH wild‐type and mutant glioblastomas using transcriptome sequencing analysis. GSEA enrichment analysis revealed that the signaling pathways activated in IDH wild‐type tumors were IFN‐α response, TNF‐α signaling via NF‐κB, IL‐6 JAK STAT3 signaling, epithelial‒mesenchymal transition, inflammatory response, and IFN‐γ response (Figure S2A). The inhibited signaling pathways were oxidative phosphorylation, Myc targets V1, and β‐catenin signaling. The box plot shows a higher IFN‐α response score in the IRF7 high‐risk group (Figure S2B). Moreover, the radar plot shows that IRF7 was associated with signaling pathways, such as IFN‐α response, IFN‐γ response, allograft rejection, coagulation, and IL‐2 STAT5 signaling (Figure S2C). Tracking tumor immunophenotype analysis revealed that the group with high IRF7 expression was highly expressed and correlated with most immune cell recruitment (Figure S2D,E). The area under the curve (AUC) value of IRF7 for the diagnosis of wild‐type and mutant IDHs was 0.817 (Figure S2F). Moreover, the prognostic analysis revealed that patients in the IRF7 high‐expression group had a worse prognosis in The Cancer Genome Atlas (TCGA) and Chinese Glioma Genome Atlas (CGGA) cohorts (*p* < 0.05) (Figure S2G,H). IRF7 expression was higher in the wild‐type IDH group than in the mutant IDH group (Figure S2I,J). IRF7 expression was also analyzed in patients with different clinical characteristics. The IRF7 high‐expression group included WHO grade II, WHO grade III, WHO grade IV, 1p19q non‐codeleted, IDH wild‐type, progressive disease and stable disease, astrocytoma, oligoastrocytoma, oligodendroglioma, and glioblastoma tumors and had a worse prognosis (*p* < 0.05) (Figure S3A‒O).

### 2.6 Identification of high transcriptional activity subtypes in IDH wild‐type gliomas and construction of a machine learning prognostic model

We used the IRF7 regulon and differential gene intersection genes as subtype typing gene sets (Table S2 and Figure S4A). Based on the previously analyzed gene set, we identified two distinct subtypes in patients with wild‐type IDH (Figure S4B). Compared to the C2 group, the prognosis of patients with C1 subtype glioma was worse (*p* < 0.05) (Figure S4C,D). Further analysis was conducted to explore the relationship between the subtypes and 13 cell death pathways. Compared with the C2 subtype, the C1 subtype displayed higher scores for alkaliptosis, apoptosis, cuproptosis, ferroptosis, necroptosis, netotic cell death, oxeiptosis, PANoptosis, parthanatos, and pyroptosis (*p* < 0.05) (Figure S4E). Autophagy and entotic cell death were the cell death pathways with higher scores in the C2 subtype (*p* < 0.05). A boxplot is used to illustrate the scores for the 13 immune features in the subgroups (Figure S4F). The C1 subtype demonstrated higher scores for antigen‐presenting cell (APC) co‐stimulation, CC chemokine receptor (CCR), checkpoint, cytolytic activity, human leukocyte antigen (HLA), inflammation‐promoting, parainflammation, T‐cell co‐stimulation, and type I IFN response (*p *< 0.05). In contrast, the C2 subtype exhibited a higher score in the type II IFN response (*p *< 0.05). Gene set variation analysis (GSVA) enrichment analysis showed that the upregulated signaling pathways in the C1 subtype were base excision repair, spliceosome, pentose phosphate pathway, fatty acid metabolism, peroxisome, apoptosis, proteasome, and riboflavin metabolism (Figure S4G). The down‐regulated signal pathways were neuroactive ligand‒receptor interaction, gap junction, calcium signaling pathway, adherens junction, O‐glycan biosynthesis, axon guidance, TGF‐β signaling pathway, glycerolipid metabolism, galactose metabolism, and ECM‒receptor interaction. Additionally, we assessed the subtype characteristics using four immune infiltration methods: CIBERSORT, EPIC, Xcell, and MCPCounter (Figure S4H). The highly infiltrating immune cells in the C2 subgroup were NK cells activated, CD4 memory resting cells, monocytes, CD8 T cells, switched memory B cells, cDC, and platelets. Highly infiltrating immune cells in the C1 subtype were cytotoxic lymphocytes, T cells, myeloid dendritic cells, and CD8+ naïve T cells. Moreover, the C1 subtype had copy number variation (CNV) mutations in most genes and a high tumor grade (Figure S4I). CNV analysis of the mutations in the C1 and C2 subtypes found that there were more mutations in the C1 subtype than in the C2 subtype. Most tumors in the C1 subtype were graded as WHO III and IV.

**FIGURE 6:**
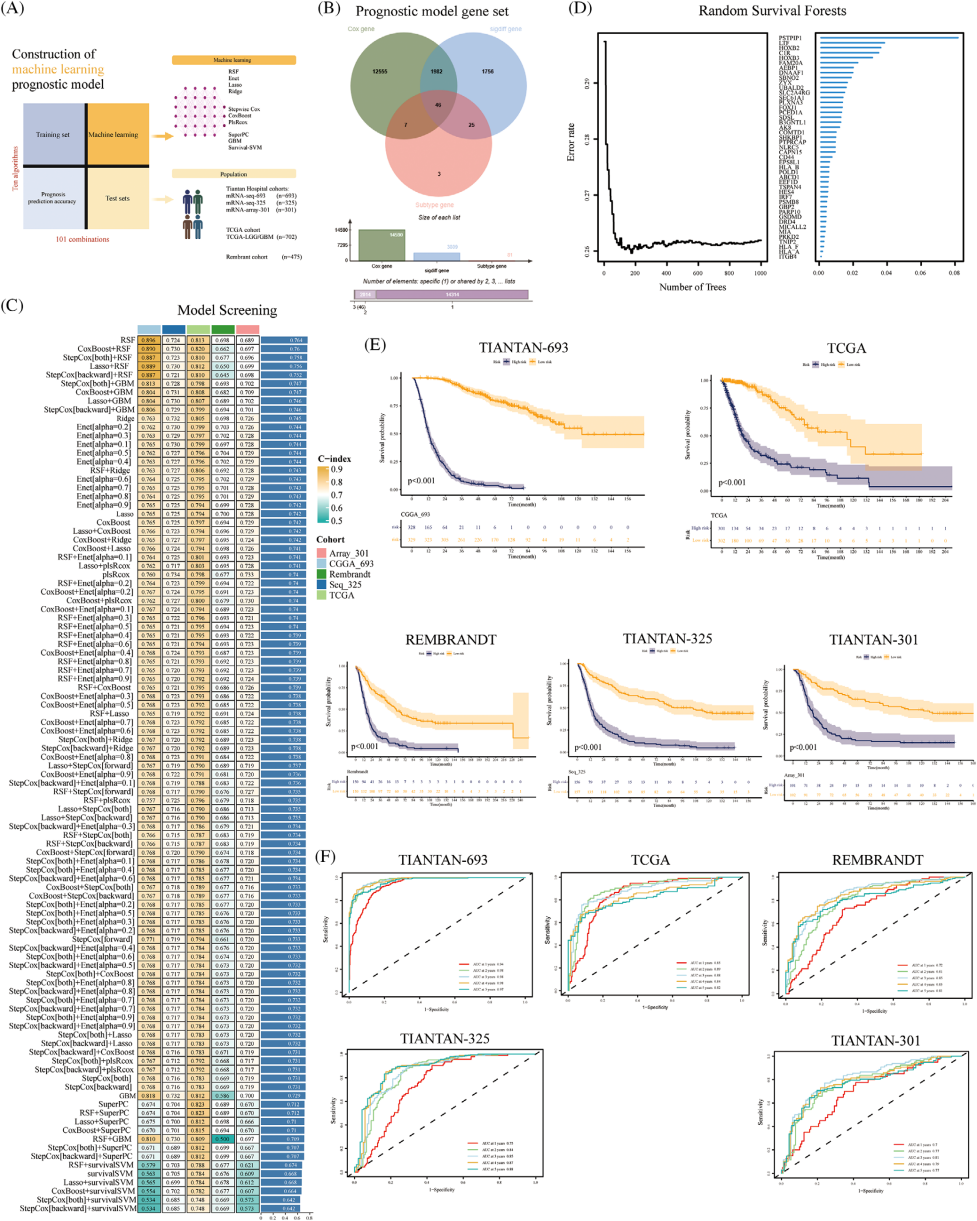
Machine learning to construct the prognosis model of glioma. (A) Prognostic model screening process roadmap. (B) The Wayne diagram showed the prognostic model input gene set screening. (C) The heatmap showed a variety of machine learning to screen the optimal prognosis model. (D) The gene screening process by random survival forests. (E) Kaplan‐Meier (KM) prognostic analysis showed the prognosis of high‐ and low‐risk groups in multiple cohorts. (F) The receiver operating characteristic (ROC) curve showed the area under the curve (AUC) values of the prediction model in different cohorts of 1, 2, 3, 4, and 5 years.

Next, we constructed prognostic models using multiple machine learning algorithms, using six clinical cohorts (Figure 6A). We used a subtype gene set, differentially expressed genes between the C1 and C2 subsets, and univariate Cox regression prognostic genes to construct a prognostic model input gene set (Table S3 and Figure 6B). The random survival forests (RSF) model was determined to be the optimal model after evaluating 101 combinations of nine machine learning algorithms. In the training set, TIANTAN‐693, the RSF model achieved a consistency index (*C*‐index) of 0.896 (Figure 6C). In the test set, the *C*‐index values were 0.724, 0.813, 0.698, and 0.689. Notably, the gene with the highest weight in the feature selection process of the RSF model was PSTPIP1 (Figure 6D). Additionally, multiple cohort‐based prognostic analyses revealed that patients with high‐risk scores had a worse prognosis (*p* < 0.05) (Figure 6E). In the TIANTAN‐693 cohort, the AUC values of the IDH wild‐type mRNA (IDHWT mRNA) model for 1−5 years were 0.94, 0.98, 0.98, 0.98, and 0.97, respectively (Figure 6F). Compared to clinical features such as IDH mutation, tissue type, and grade, the IDHWT mRNA model exhibited a higher *C*‐index (Figure S5A). Furthermore, compared with several previously published prognostic models, the IDHWT mRNA model consistently demonstrated favorable results (Figure S5B and Table S4).

### 2.7 Biological significance of modeled risk populations

**FIGURE 7:**
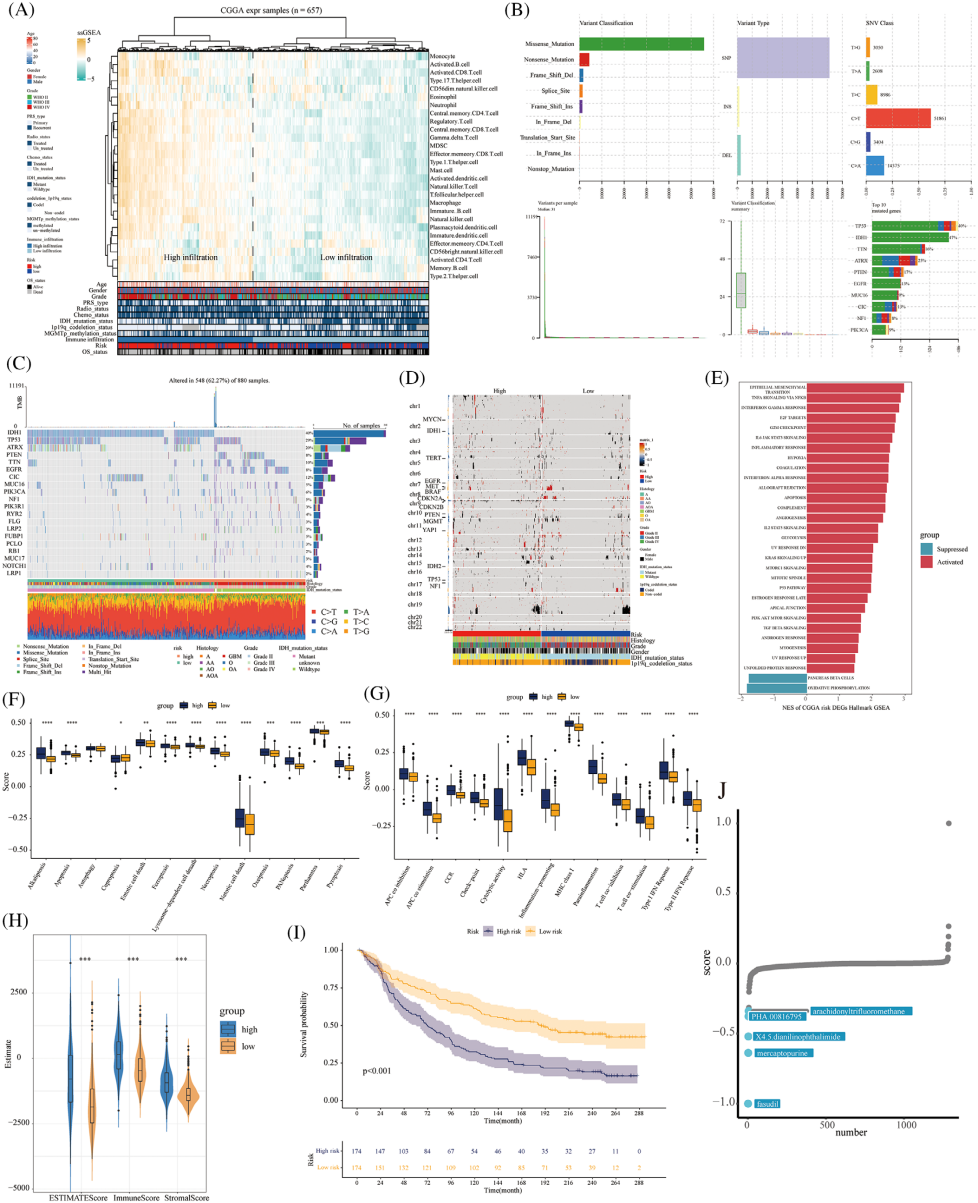
The biological significance of model patients in high‐ and low‐risk groups. (A) The heatmap showed the relationship between high‐ and low‐risk groups, clinical features, and immune cell infiltration. (B) The histogram showed glioma variant classification, variant type, Single‐nucleotide variant (SNV) class, variants per sample, variant classification summary, and top 10 mutated genes. (C) The heatmap showed gene mutations in high‐ and low‐risk groups. (D) The heatmap showed the copy number alterations score of patients in high‐ and low‐risk groups. (E) The histogram showed the signal pathway enrichment analysis of differential genes in high‐ and low‐risk groups. (F and G) The box chart showed 13 cell death scores and immune function scores of patients in high‐ and low‐risk groups. (H) The violin picture showed the ESTIMATE score, immune score, and stromal score of the high‐ and low‐risk groups. (I) Prognostic KM was used to analyze the prognosis of the high‐ and low‐risk groups in the imvigor210 immunotherapy cohort. (J) Top five small molecule drugs likely to act on patients in high‐risk groups. ^*^ *p* < 0.05, ^**^ *p* < 0.01, ^***^ *p* < 0.001.

We screened prognostic model genes to construct high‐ and low‐risk groups. The heatmap shows the relationship between immune cell infiltration, clinically related indicators, and risk population. Most high‐risk patients had high immune cell infiltration, WHO IV, wild‐type IDH, and 1p19q non‐deletion (Figure 7A). The most common variant classification of gliomas is based on missense mutations. The 10 most commonly mutated genes were *TP53*, *IDH1*, *TTN*, *ATRX*, *PTEN*, *EGFR*, *MUC16*, *CIC*, *NF1*, and *PIK3CA* (Figure 7B). Compared with patients in the low‐risk group, those in the high‐risk group had fewer mutations in *IDH*, *TP53*, *ATRX*, and *CIC*. In addition, the main mutations in high‐risk patients were in *PTEN*, *TTN*, and *EGFR* (Figure 7C). The heatmap showed that the copy number for patients in the high‐risk group varied greatly, and most had WHO grade IV, wild‐type IDH, and 1p19q non‐deletion (Figure 7D). GSEA showed that the activated signal pathways in the high‐risk group were epithelial‒mesenchymal transition, TNF‐α signaling via NF‐κB, IFN‐γ response, E2F targets, and G2M checkpoint. The inhibited signal pathways were pancreas β‐cells and oxidative phosphorylation (Figure 7E). After comparison of the 13 modes of death scores, high‐risk patients scored higher for alkaliptosis, apoptosis, entotic cell death, ferroptosis, lysosome‐dependent cell death, necroptosis, netotic cell death, oxeiptosis, PANoptosis, parthanatos, and pyroptosis (*p* < 0.05) (Figure 7F). Moreover, compared to patients in the low‐risk group, high‐risk patients had higher expression of 13 immune functions, including APC co‐inhibition, APC co‐stimulation, CCR, checkpoint, cytolytic activity, HLA, inflammation promoting, major histocompatibility complex (MHC) class I, parainflammation, T‐cell co‐inhibition, T‐cell co‐stimulation, type I IFN response, and type II IFN response (*p < *0.05) (Figure 7G). Meanwhile, patients in the high‐risk group had higher ESTIMATE, immune, and stromal scores (*p* < 0.05) (Figure 7H). Prognosis was worse in the high‐risk group than in the low‐risk group in the immunotherapy cohort (*p* < 0.05) (Figure 7I). The CMAP Drug Database predicted that the five top‐ranked small‐molecule candidates for high‐risk patients were arachidonyl trifluoromethane, PHA.00816795, 4,5‐dianilinophthalimide, mercaptopurine, and fasudil (Figure 7J).

### 2.8 Experimental validation of the biological role of IRF7

**FIGURE 8:**
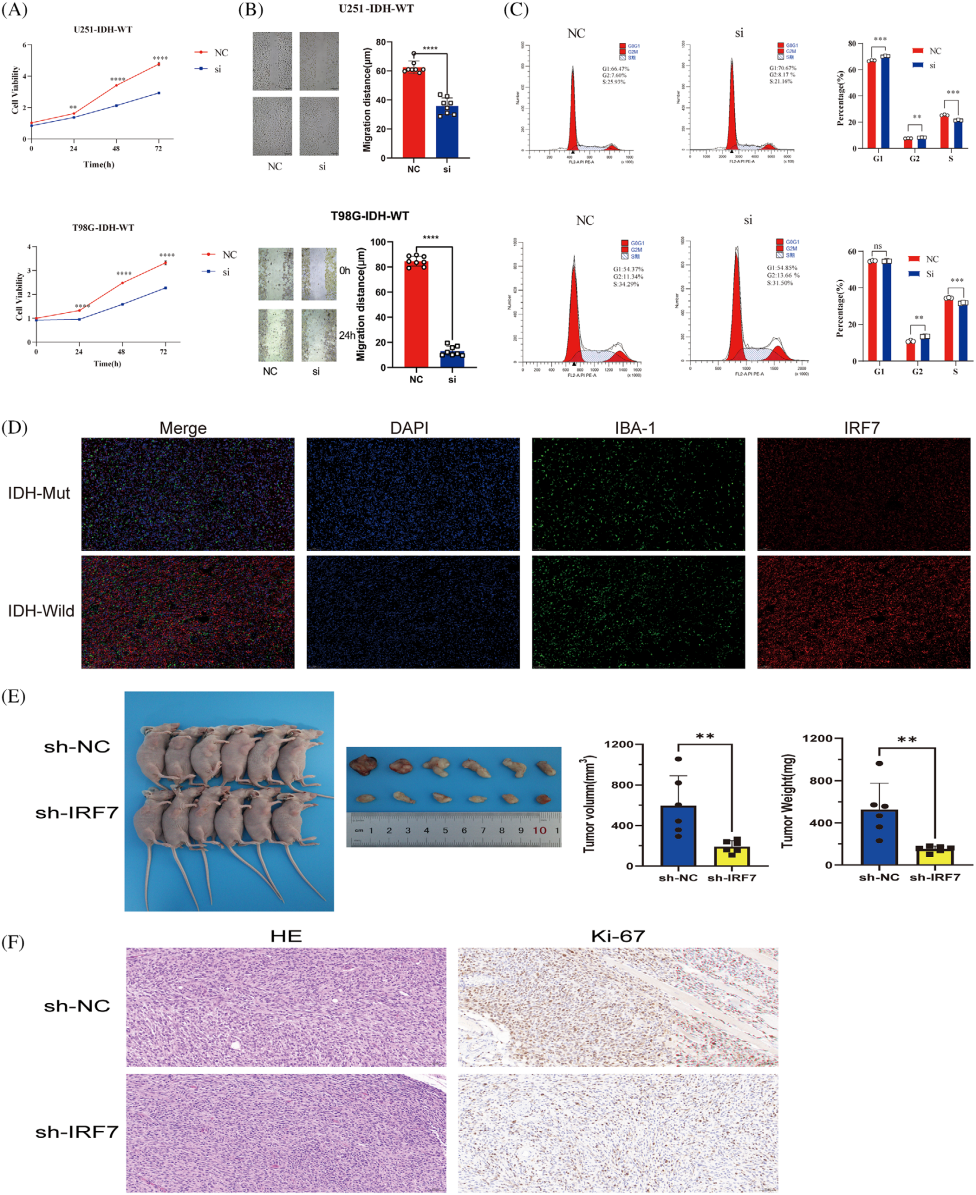
IRF7 promotes T98G and U251 cell viability and migration. T98G and U251 were transfected with IRF7‐siRNA and negative control (NC), respectively. (A) Cell viability was measured by the CCK‐8 assay in T98G and U251, separately. (B) Cell migration was measured by the wound healing assay in T98G and U251, separately. (C) Cell cycle was measured by the flow cytometry assay in T98G and U251, separately. Each experiment was repeated three times with similar results. (D) Confocal (IF microscopy indicated strong IRF7 immunofluorescent staining in IDH‐wild glioma tissues by merging DAPI and IBA‐1, which is a marker of activated microglia and inflammation. (E) Representative images of subcutaneous tumors formed by sh‐NC and sh‐IRF7 glioma cells. Tumor volume and weights measurements after excision, presented as mean ± SEM, showing significant differences using Student's *t*‐test. (F) Representative hematoxylin and eosin (HE) staining images showing the histological features of the tumors. Representative Ki‐67 immunohistochemical staining images indicating the proliferative activity in the tumors. Quantification of Ki‐67‐positive cells is presented as mean ± SEM. ^*^ *p* < 0.05, ^**^ *p* < 0.01, ^***^ *p* < 0.001.

To further validate the potential oncogenic role of IRF7 in gliomas, Cell Counting Kit‐8 (CCK‐8) and wound healing assays were performed using U251‐IDH‐WT and T98G‐IDH‐WT cell lines. An siRNA sequence against IRF7 was designed and assessed for its knockdown efficiency in U251‐IDH‐WT cells. The CCK‐8 assay was performed to assess the role of IRF7 in glioma cell proliferation. Downregulation of IRF7 resulted in a significant decrease in OD450 values in U251‐IDH‐WT and T98G‐IDH‐WT cells 48 h after transfection (*p < *0.05) (Figure 8A). To assess the function of IRF7 in cell migration, a wound healing assay was performed, which showed that knockdown of IRF7 significantly decreased the wound healing rate in both U251‐IDH‐WT and T98G‐IDH‐WT cells at 24 h, suggesting that downregulation of IRF7 could inhibit the migration of glioma cells in vitro (*p *< 0.05) (Figure 8B).

The cell cycle distribution of U251‐IDH‐WT and T98G‐IDH‐WT cells treated with the siRNA sequence against IRF7 was analyzed by comparing the percentages of cells in the G0/G1, S, and G2/M phases, without considering apoptotic cells. As shown in Figure 8C, after treatment with the siRNA sequence against IRF7, the percentage of cells in the G1 and S phases significantly increased, and the percentage of cells in the G2/M phase decreased. These results indicated that the siRNA sequence against IRF7 promoted the arrest of cells in the G1 and S phases of the cell cycle, thereby inhibiting U251‐IDH‐WT and T98G‐IDH‐WT cell proliferation.

We assessed the density and distribution of IRF7 and IBA1 in formalin‐fixed, paraffin‐embedded tumor sections using immunofluorescence microscopy coupled with quantitative digital imaging. Immunofluorescence staining revealed distinct patterns of IRF7 and IBA1 expression in IDH‐negative and IDH‐positive glioma tissues. Notably, an upregulation of IRF7 was observed in the IDH‐negative glioma tissues, which were frequently encircled by macrophages or microglia (Figure 8D). We established a subcutaneous xenograft model to assess the role of IRF7 in glioma progression in vivo. Xenografts from IRF7‐knockdown U251 cells showed reduced growth compared to those in the control group. Post‐excision, IRF7 xenografts weighed less and were smaller than their control counterparts, indicating that IRF7 potentially promoted tumor effects by impeding glioma growth (Figure 8E). Ki67 immunohistochemistry and hematoxylin‒eosin (HE) staining were performed on the excised tumors, revealing that the sh‐IRF7 group exhibited markedly lower Ki67 expression and less aggressive histological features as indicated by the HE staining, compared to the sh‐NC group. These results suggest that IRF7 knockdown effectively suppresses tumor growth and proliferation in this model (Figure 8F).

For RNA sequencing, we used U251‐IDH‐W cells with IRF7 knockdown and U251‐IDH‐W cells without IRF7 knockdown. Boxplot graphs demonstrate that IRF7 expression was higher in the normal group than in the knockdown group (Figure S6A). Moreover, through GSVA enrichment analysis, it was found that the upregulated signaling pathways in the knockout cell lines were adherens junction, tight junction, one‐carbon pool by folate, pentose phosphate pathway, nucleotide excision repair, and nitrogen metabolism (Figure S6B). Downregulated signaling pathways included biosynthesis of unsaturated fatty acids, glyoxylate and dicarboxylate metabolism, GnRH signaling pathway, the citrate cycle, melanogenesis, pyrimidine metabolism, the JAK‒STAT signaling pathway, and sphingolipid metabolism. At the same time, GSEA enrichment analysis showed that the signal pathways inhibited were E2F targets, IFN‐α response, IFN‐γ response, Myc targets v1, G2M checkpoint, mTORC1 signaling, and TNF‐α signaling via NF‐κB (Figure S6C).

## 3 DISCUSSION

IDH serves as the most important biomarker for glioma prognosis. A comprehensive understanding of the differences in transcriptional regulation between wild‐type and mutant IDH gliomas has significant implications for their treatment. We used single‐cell sequencing to reveal differences in transcriptional regulation between different cellular subpopulations of IDH‐mutant and wild‐type gliomas. Moreover, transcriptionally regulated genes were found to exhibit high transcriptional activity in the microglia and macrophages of wild‐type IDH mice. Among these, IRF7 is involved in microglial subpopulations via IFN‐related signaling pathways. We used consistency clustering of the wild‐type IDH subtypes. Subsequently, we used various machine learning algorithms to screen and construct a glioma prognostic model centered on IRF7 target genes. These findings contribute to a better understanding of the mechanisms underlying glioma development and provide a theoretical foundation for personalized treatment strategies.

The immune microenvironment plays a crucial role in the development and progression of gliomas, influencing key processes, such as tumor cell growth, invasion, and immune evasion. Our study revealed differences in cellular subpopulations of the immune microenvironment between wild‐type and mutant IDH tumors. In wild‐type IDH tumors, astrocytes, microglia, and oligodendrocytes accounted for 58.2%, 18.4%, and 5%, respectively, of cells in the TME. In the IDH‐mutant tumors, astrocytes, microglia, and oligodendrocytes accounted for 34.6%, 33%, and 20.5%, respectively. Neural stem cell features include neural stem cell genes and markers of astrocytes.^15^ Astrocytes are the most abundant cells in the central nervous system (CNS). They perform essential functions during development and homeostasis in vivo, such as participation in the maintenance of the blood‒brain barrier, storage, distribution of energy substrates to neurons, and support for the development of neuronal cells and synapses.^16^, ^17^ The interaction between astrocytes and the resident or infiltrating cells of the CNS plays a significant role in tissue physiology and pathology.^18^ Studies have shown that the depletion of astrocytes inhibits the growth of gliomas and drives the pathogenicity of glioblastomas by regulating the immune metabolism of the TME.^19^ This may explain the higher proportion of astrocytes in the wild‐type IDH microenvironment than in the mutant IDH microenvironment.

Our study found that the most highly active TFs were concentrated in IDH wild‐type microglial cells and macrophages. The glioblastoma microenvironment consists of brain‐resident microglial cells and infiltrating monocytes, which differentiate into macrophages once they infiltrate the tumor.^20^, ^21^ The CNS immune defense is composed of microglial and small glial cells that serve as innate immune sentinels and frontline host immune barriers against pathogenic insults.^22^, ^23^ Moreover, these myeloid lineage cells constitute the major immune population in gliomas, accounting for 30%−50% of the total cellular composition.^24^, ^25^, ^26^ Tumor‐associated macrophages in early glioblastomas consist of single microglial cells that evolve into a mixture of microglia, infiltrating monocytes and macrophages.^27^ Microglia in the CNS have different phenotypes, among which M2 microglia produce immunosuppressive cytokines and promote glioma cells.^28^ In the glioma TME, microglia show high oxidative stress and decreased antigen presentation ability, resulting in a significant decrease in the number and function of CD8+ T cells, inducing the formation of an immunosuppressive microenvironment, thus promoting the growth and progress of the tumor.^29^ In our study, microglial cells accounted for much lower proportion of cells in the TME in IDH wild‐type tumors than in IDH‐mutant tumors.

The interferon regulatory factor (IRF) family comprises nine members that act as TFs and play various roles in inflammatory responses, including antiviral defense, cell proliferation, and immune cell maturation.^30^, ^31^, ^32^ Previous studies have demonstrated that IRF7 is a critical determinant of glioma progression and heterogeneity of glioblastoma.^33^ Inflammatory signaling mediated by IRF7 promotes neural glioma stem cells and angiogenesis and serves as a major driving force in the progression and cellular heterogeneity of brain tumors.^33^ The expression of IRF7 increases during the M2 to M1 transition in vitro and in vivo in microglial cells. Knockdown of IRF7 using siRNA inhibited the expression of M1 marker mRNA and reduced the phosphorylation of STAT1.^34^ While PSTPIP1 emerged as the gene with the highest weight in the feature selection process of the RSF model, our focus on IRF7 was driven by its consistent role across various analyses. IRF7 was not only differentially expressed but also involved in crucial signaling pathways and immune responses specific to IDH wild‐type gliomas. Its potential impact on the TME and patient prognosis, as well as its involvement in immune modulation, made it a prime candidate for further exploration and validation in our study. We acknowledge that PSTPIP1's role warrants further investigation, and future studies could explore its potential as a prognostic biomarker or therapeutic target in gliomas.

Our study found that TFs in IFN‐related signaling pathways were enriched and are mostly found in microglia and macrophages. IRF proteins, commonly IRF1, 2, and 3, influence the production and secretion of cytokines. A randomized, multicenter, phase III clinical trial demonstrated the efficacy of TMZ in combination with IFN‐α in the treatment of newly diagnosed high‐grade gliomas.^35^ In patients with unmethylated methylguanine methyltransferase (MGMT), who are insensitive to radiation and chemotherapy, TMZ + IFN treatment significantly improved the overall survival time from 17.40 to 24.67 months compared to TMZ alone.^35^ IRF7 is located downstream of pattern recognition receptor‐mediated signaling pathways and is critical for type 1 IFN production. IRF7 is expressed in the spleen, lymph nodes, and bone marrow, particularly in epithelial cells, monocytes, and macrophages,^36^ and promotes tumor growth in some cancers.^34^, ^37^, ^38^ IRF7 activation can induce tumor cells to produce IFNs, activate immune cells, and enhance antitumor immune responses. However, gliomas have an immunosuppressive microenvironment that includes immunosuppressive factors derived from tumor cells, depleted cytotoxic T lymphocytes, regulatory T cells, and downregulated MHC molecules.^39^ These factors may inhibit the activity of immune cells, weaken the immune regulatory role of IRF7, and consequently lead to poorer prognosis. Moreover, high IRF7 expression may be associated with wild‐type gliomas, which typically have a worse prognosis. By integrating bulk RNA‐seq with scRNA‐seq data, we have further delineated the role of IRF7 in IDH wild‐type gliomas. Our integrative analysis shows that IRF7 is a key regulator within the IFN signaling pathway, predominantly influencing microglial and macrophage subpopulations. This integration allowed us to confirm that IRF7 not only drives the transcriptional reprogramming in these immune cells but also correlates with worse prognostic outcomes, underscoring its potential as a therapeutic target. Furthermore, the spatial transcriptome data provided insight into the specific localization of IRF7 expression, suggesting that its effects are most pronounced in the central regions of the tumor, where immune suppression and tumor growth are actively regulated.

In patients with wild‐type IDH gliomas, the median survival is only 14 months. We used regulons of IRF7 to construct the molecular subtypes of patients with wild‐type IDH. The C2 subtype had a worse prognosis, with higher scores for various cell death pathways and immune cell features. A high immune cell feature score may reflect increased infiltration and activity of immune cells. However, this increase in immune cells may be combined with immune escape mechanisms and an immunosuppressive microenvironment, leading to poor prognosis. Cell death is an important factor in tumor development and response to treatment. A high score for cell death pathways may reflect the increased proliferation and invasion capabilities of tumor cells, as well as resistance to treatment. This may contribute to tumor progression and a worsened prognosis. Therefore, further research is needed to validate these findings and gain a deeper understanding of the mechanisms underlying IRF7 regulation in IDH wild‐type gliomas. In addition, this study provides a new avenue for predicting the prognosis of patients with wild‐type IDH.

We also constructed a prognostic model for glioma IDHWT mRNA expression. The RSF was identified as the optimal algorithm. Moreover, the prognostic difference between patients in the high‐ and low‐risk groups of the model was significant. The *C*‐index of the model was high for both the training and test sets, with an average of 0.764. Furthermore, the AUC values of the 1‐, 2‐, 3‐, 4‐, and 5‐year receiver operating characteristic (ROC) curves reached 0.9. The stable performance of IDHWT mRNA features in both the training and test sets suggests that IDHWT mRNA features have great potential for clinical applications. The classical molecular markers IDH, 1p/19q, and MGMT status have long been used to evaluate clinical strategies and prognosis in patients with glioma.^40^, ^41^ However, the IDHWT mRNA signature was significantly superior to these markers and is an independent risk factor. Comparative analyses of the 95 published and publicly available prognostic models of the trait showed that the C‐index of our model was superior to that of most of the cohorts. This reinforces the potential value of this study.

Although our study included single‐cell sequencing, transcriptome sequencing, and in vivo experiments, some limitations need to be noted. First, the single‐cell datasets for IDH‐mutant and wild‐type blood and tumor tissue samples were derived from a single source. Additionally, while we recognize the importance of single‐cell spatial transcriptome data for IDH‐mutant gliomas, which would provide valuable insights into their spatial organization and gene expression patterns, we were unable to access such data during our study. Future studies should validate our findings using multiple datasets and incorporate single‐cell spatial transcriptome analysis for IDH‐mutant gliomas to better understand their spatial heterogeneity and gene expression profiles. Second, all included datasets were from retrospective cohort studies, and the IDHWT mRNA signature prognostic model should be validated in a prospective multicenter cohort. Third, although some IRF7 cellular mechanistic studies have been conducted, further mechanistic validation in microglial cell lines and with a glioma TME is needed. Finally, this study focused on the initial exploration of gene transcriptional regulation in wild‐type and mutant IDH tumors, which should be further investigated in the future in combination with chromatin immunoprecipitation‐sequencing.

## 4 CONCLUSION

Our study targeted the GRN of IDH‐mutant and wild‐type gliomas for a comprehensive analysis. The IFN‐associated signaling pathway, IRF7, and its regulons potentially act on IDH wild‐type microglia and macrophage subpopulations. Clinical subtypes of wild‐type IDH gliomas were identified. Finally, based on extensive machine learning algorithms, a stable and robust IDHWT mRNA signature was developed to predict the prognosis of patients with glioma.

## 5 METHODS

### 5.1 Study design and data sources

The scRNA‐seq data from tumor tissues were sourced from the European Genome‐Phenome Archive database (EGAS00001005300).^9^ There were six IDH‐mutant and five IDH wild‐type glioma tissue samples. Peripheral blood mononuclear cell (PBMC) scRNA‐seq samples from patients with glioma, including IDH‐mutant (*n* = 2) and wild‐type IDH (*n* = 3), were obtained from Beijing Tiantan Hospital. These data were deposited in the Gene Expression Omnibus (GEO) dataset (GSE237779). Single‐cell spatial transcriptome data, from IDH wild‐type glioblastoma (*n* = 3), secondary glioblastoma (*n* = 2), and glioblastoma paraneoplastic tissue (*n* = 1), were obtained from the West China Hospital of Sichuan University. The data were stored in the GEO dataset (GSE194329).

We utilized clinical information and transcriptomic data from the GEO, Beijing Tiantan Hospital, and TCGA databases. The datasets used in this study included TIANTAN‐693 (*n* = 693), TIANTAN‐325 (*n* = 325), TIANTAN‐301 (*n* = 301), TCGA‐LGG/GBM (*n* = 702), and REMBRANDT (*n* = 475), comprising a total of 2496 patients. Some of the data were obtained from the CGGA database (Table S5).

### 5.2 Data processing

During data processing, a minimum gene count of 200, a maximum gene count of 7000, and a ribosomal count of less than 5% were set. In total, 2000 highly variable genes were identified. The R package “harmony” was used to reduce batch effects, as well as to reduce biases between different patients. Manual cell type annotation was performed based on previous literature and the Cellmarker database, including astrocyte (S100B, AQP4, GFAP), microglia (CSF1R, CX3CR1, P2RY12, TMEM119), oligodendrocyte (OLIG1, MAG, OLIG2, PLP1, MOG, MBP), epithelial (CD44, CDH2), macrophage (AIF1, CD163, CD68), neuron (DCX, MAP2, STMN2), T cell (CD3D, CD3E, CD8A), and endothelial cell (VWF, CD34, FLT1, CLDN5).^9^, ^42^, ^43^, ^44^ Visualization was performed using the R package “SCP.”

### 5.3 TF gene regulation analysis and visualization

SCENIC is a method for reconstructing GRNs and identifying cell states based on co‐expression and motif analyses of single‐cell transcriptomic data.^45^ The human TF gene set was obtained from the cisTarget database (https://resources.aertslab.org/cistarget). The GRN and cisTarget were constructed using pySCENIC version 9.18 and version 12.1, respectively. The meta cell matrix of IDH wild‐type and IDH‐mutant single cells was first constructed to speed up the efficiency of subsequent operations. The meta cell method is a technique used to aggregate individual cells into meta cells based on their similarity in gene expression profiles. This aggregation helps reduce the computational burden and improves the efficiency of downstream analyses. In our study, we obtained meta cells using a two‐step process. First, we performed dimensionality reduction on the single‐cell RNA sequencing data using principal component analysis (PCA) or t‐distributed stochastic neighbor embedding. This step reduced the high‐dimensional gene expression data into a lower‐dimensional space, capturing the major sources of variation. Next, we applied a clustering algorithm, such as *k*‐means or hierarchical clustering, to group the cells based on their reduced‐dimensional representation. The resulting clusters represented the meta cells, which were then used for subsequent analyses. Co‐expression networks were obtained by gene co‐expression analysis to establish possible TF‐target regulatory relationships. The minimum number of regulon genes was set to 10. The target genes of regulatory factors were scored by AUCell and the regulon activity score (RAS) of the regulators was calculated, and then the cells were downscaled and categorized according to their activities using UMAP and PCA methods. ANOVA was used to find different groupings and cell‐specific TFs.

To understand regulatory graph based clustering. import TF‐target credibility (valued by feature importance), merge regulatory information and relevance information, and count the targets of each TF. A cis‐regulatory motif analysis was performed for each co‐expression module using RcisTarget. Binding motifs of TFs significantly enriched in the gene list were identified. AUCell scored the activity of each regulon in each cell and calculated the RAS.^10^ UMAP plots were used to visualize the distribution of IDH wild‐type and IDH‐mutant RAS in subpopulations of cells. The correlation of RAS between different subpopulations was calculated. Next, calculate the mutual Connection Specificity Index. hierarchical clustering results were processed the specified height threshold h was 8 and the clustering results were cut from the dendrogram. Visualize the clustering results by heatmap. The average activity of each TF module was plotted and the individual modules were visualized using UMAP plots.

### 5.4 Single‐cell sequencing of glioma peripheral blood mononuclear cells

To further analyze the expression of IRF7 in glioma PBMC, we used data from five patients from Beijing Tiantan Hospital with gliomas, two with IDH mutations and three with wild‐type IDH. The cell annotation process was based on the integrated file of the original article.^44^ The wild‐type and mutant signal pathways of IDH were analyzed by R packet “SCP” and GSEA enrichment.

### 5.5 Single‐cell spatial transcriptome analysis of key gene IRF7 expression

To further demonstrate the spatially localized expression of IRF7 and the IRF7 regulon in glioma tissues, we used six glioma tissues for spatial transcription analysis. Genes expressed at less than 10 points were screened by filtering. PCA was used to reduce the clustering dimensions. The visualization process performed data visualization using DimPlot and SpatialDimPlot. To further annotate tumor cell and non‐malignant cell features in the spatial transcriptome, we assessed tumor cell content in different glioma tissues.^46^, ^47^

### 5.6 Subtype identification of IDH wild‐type gliomas with high transcriptional activity signature

To further identify the subtypes in patients with IDH wild‐type gliomas, we intersected the regulon genes of IRF7 with differentially expressed genes in the tumor tissue. The criteria for differential gene selection were set as adj.P.Val < 0.05 and logFC > 1. Consensus clustering was performed to classify subtypes of patients with wild‐type gliomas. Single‐sample gene set enrichment analysis (ssGSEA) was used to score the subtypes based on 13 different death pathways and immune features.^48^, ^49^ Additionally, we investigated the differences in CNV and clinical characteristics between the subtypes. Finally, survival curves for the subtypes were plotted using the “survival” and “survminer” R packages.

### 5.7 Constructing a prognostic model by multiple machine learning algorithms

The gene set used for model construction consisted of the intersection of glioblastoma univariate Cox prognostic, differential, and subtype genes. Furthermore, we employed nine machine‐learning algorithms in 101 combinations.^50^, ^51^ The integrated machine learning algorithms included RSF, elastic networks (Enet), Lasso, Ridge, stepwise Cox, CoxBoost, partial least squares regression for Cox (plsRcox), supervised principal components (SuperPC), generalized boosted regression modeling (GBM), and survival support vector machine. The TIANTAN‐693 cohort was used as the training cohort, whereas the TIANTAN‐325, TIANTAN‐301, REMBRANDT, and TCGA cohorts were used as the test cohorts. Survival curves were plotted for patients in the high‐ and low‐risk groups. Diagnostic ROC curves were plotted for 1−5 years. Subsequently, the *C*‐index was calculated to compare the model signature (IDHWT mRNA) with clinical features. Additionally, we compared the *C*‐index of our model with those of 65 previously published prognostic models.

### 5.8 Biological differences in patients with high‐ and low‐risk IDH wild‐type mRNA signature

We evaluated the biological differences between patients in the high‐ and low‐risk groups. For the classified patients, immune cell infiltration and clinical features were compared using ssGSEA. We also compared CNV between the two groups.^52^ Using ssGSEA, we compared the scores for 13 types of cell death and 13 immune cell features in patients in the high‐risk group.^49^, ^53^ Additionally, to identify potential drugs that could counteract the high‐risk phenotype, we utilized the Connectivity Map (CMap) database, which includes the gene expression profiles of 1309 drugs after the treatment of five cell lines. We selected the top 300 genes with high and low expression levels for CMap analysis by comparing the gene expression profiles of high‐ and low‐risk patients. A lower score indicated a higher likelihood of the drug reversing the molecular features of the disease.

### 5.9 Cell culture and transfection

U251‐IDH‐WT and T98G‐IDH‐WT cells were cultured in Dulbecco's modified Eagle's medium supplemented with 10% fetal bovine serum (FBS) and maintained at 37°C in 5% CO_2_. Gene‐specific and negative control siRNAs were purchased from RiboBio. NC‐siRNA: 5′‐UUCUCCGAACGUGUCACGUTT‐3′, IRF7‐siRNA: TCGAGTGCTTCCTTATGGA. The Ribo FECTTM CP Transfection Kit was used to transfect siRNAs into six‐well plates when the cell density reached approximately 50%. The cells were harvested for subsequent treatments 24 h after transfection.

According to the human IRF7 gene sequence available on the NCBI website, validated IRF7‐shRNA sequences were selected using Sigma's online tool (https://www.sigmaaldrich.cn/CN/zh/semi‐configurators/shrna?activeLink=productSearch). The shRNA sequence synthesized by Sangon Biotech Co., Ltd. was sh‐IRF7: GCTGGACGTGACCATCATGTA. When the U251 cell density reached 50%−60%, the cells were infected with lentivirus (MOI = 10) and divided into negative control (NC) and IRF7‐shRNA groups. Then, 24 h post‐infection, the medium was replaced with a virus‐free complete medium. Expression of green fluorescent protein in the cells was observed under a fluorescence microscope 48−72 h later. Puromycin (2.5 µg/mL) was added to select stably transfected clones for subsequent experiments.

### 5.10 CCK‐8 proliferation assay

The CCK‐8 assay was used to evaluate cell proliferation. U251‐IDH‐WT and T98G‐IDH‐WT cells were seeded in 96‐well plates at a density of 5000 cells/100 µL/well and treated for 24−72 h. Then, 10 µL of CCK‐8 solution (Vazyme, A311‐01) was added to each well of the plates and incubated for 0.5 h at 37°C. Finally, absorbance was analyzed at 450 nm using a Multiskan SkyHigh Thermo Scientific spectrophotometer.

### 5.11 Wound healing assay

A wound healing assay was performed to evaluate cell migration activity. U251‐IDH‐WT cells were seeded in six‐well plates at a density of 500,000 cells/1 mL/well. After 6 h, a 10 µL disposable pipette tip was run over the surface of the cells to create scratches. The cells were washed three times with phosphate‐buffered saline (PBS) and cultured in medium containing 5% FBS. The extent of wound healing was measured at 0 and 24 h.

### 5.12 Flow cytometric cell cycle analysis

To evaluate the presence of DNA fragments, U251‐IDH‐WT cells were seeded in six‐well plates at a density of 500,000 cells/1 mL/well for 24 h, harvested by trypsinization, flushed with complete culture medium, washed with PBS, and gently resuspended in 500 µL of hypotonic fluorochrome solution (450 µL of propidium iodide and 50 µL of RNase A) to stain cell nuclei. DNA content was determined using CytoFLEX (Beckman) and analyzed using ModFit software. For each sample, 10,000 events were collected, and cell cycle distribution was determined based on DNA content.

### 5.13 Tumor tissue immunofluorescence

In this study, immunofluorescence staining was performed on paraffin‐embedded tissue sections to detect the expression of the macrophage markers IRF7 and IBA1. The sections were dewaxed in water and prepared for subsequent staining. Antigen retrieval was conducted in citrate buffer (pH 6.0) in a pressure cooker placed on an induction cooker, heated until steam emission was reached, and maintained for 2 min. Following natural cooling and depressurization, the slides were oscillated in PBS (pH 7.4) on a decolorizing shaker for 5 min three times in succession to wash off excess reagents. To inhibit the activity of endogenous peroxidases, sections were incubated at room temperature in 3% H_2_O_2_ for 10 min. Rinsing was performed three times with PBS and distilled water for 3 min each. Fluorescently‐labeled antibodies specific to IRF7 (Proteintech, 22392‐1‐AP) and IBA1 (Proteintech, 10904‐1‐AP) (dilution 1:25) were then applied and incubated in a 37°C water bath for 1 h. After incubation, the sections were washed six times with PBS and distilled water for 3 min each. Subsequently, the slides were air‐dried with cold air and mounted using a glycerol‐based mounting medium. The mounted sections were stored at 4°C until examination. Finally, fluorescence microscopy was used to observe the stained sections and assess the localization and distribution of immunoglobulins.

### 5.14 Library preparation and sequencing

We used 3‐to‐3 knockout IRF7 U251‐IDH‐WT cells and non‐knockout U251‐IDH‐WT cells for RNA sequencing. RNA concentrations were quantified using a Qubit (Thermo Scientific) RNA Assay Kit with a Qubit 2.0 Fluorometer. RNA integrity was evaluated using the RNA Nano R1000 Assay Kit on a Bioanalyzer 4200 system. The criteria for constructing the database included a minimum RIN value of 7.5, RNA content of at least 50 µg, and a concentration of 3.5 µg/µL or higher. Following qualitative and quantitative assessment of total RNA, ribosomal RNA was depleted. A library was constructed using a VAHTS Universal V8 RNA‐Seq Library Prep Kit (Vazyme, NRM605‐01/02). Adaptors were then ligated to adenylated DNA fragments, followed by the purification and size selection of cDNA fragments, primarily ranging from 350 to 400 bp. Sequencing was performed using an Illumina NovaSeq X platform with a paired‐end read length of 150 bp (PE150).

### 5.15 Subcutaneous xenograft model in nude mice

In the xenograft study, female athymic nude mice (*n* = 6/group) were acclimated for 1 week. U251 cells at 80% confluency were trypsinized after fresh medium exposure for 24 h and inoculated at 5 × 10^6^ cells in 100 µL volumes into mice of each group. On day 25, the tumor volume was measured using calipers and calculated as ½ × (length × width × height), and mice were euthanized for tumor fixation in formalin and paraffin embedding. Tissue sections were stained with HE for proliferative analysis under a microscope.

## AUTHOR CONTRIBUTIONS

J.L. was responsible for the idea, writing, visualization, and revision of the article. S.L. was responsible for the experimental validation of the cells. Z.Y. was responsible for the guidance of the article and graphing. W.W., S.Y., and Q.L. were responsible for the data verification and revision of the article. X.H. was responsible for the experimental design idea. X.L. and Y.W. were responsible for the idea and data collection of the article. All the authors have read and approved the final manuscript.

## CONFLICT OF INTEREST STATEMENT

The authors declare that they have no conflicts of interest.

## ETHICS STATEMENT

The study was approved by the Institutional Ethics Committee and Institutional Review Board of Beijing Tiantan Hospital (KYSQ2021740101), and all participants signed an informed consent form. The animal experiments took place in the SPF Animal Laboratory at Zhongnan Hospital of Wuhan University (ZN2023021).

## Supporting information



Supporting Information

## Data Availability

The single‐cell sequencing of IDH‐mutant and IDH wild‐type gliomas from publicly available datasets. The scRNA‐seq data from tumor tissues were sourced from the European Genome‐Phenome Archive database (EGAS00001005300). The spatial transcriptome data were sourced from West China Hospital of Sichuan University and have been stored in the GEO database (GSE194329). Glioma blood single‐cell sequencing data were obtained from Tiantan Hospital and have been stored in the GEO database (GSE237779). The rest of the transcriptome data were from the CGGA database.
