# Supplementary material for: Single‐cell transcriptomics reveals IRF7 regulation of the tumor microenvironment in isocitrate dehydrogenase wild‐type glioma
Source: MedComm (2020). 2024 Nov 3;5(11):e754. doi: 10.1002/mco2.754 (PMC11531655; doi:10.1002/mco2.754)
Supplement: Supplementary file 1 — Supporting Information [file MCO2-5-e754-s001.docx]

**Single-cell Transcriptomics Reveals IRF7 Regulation of the Tumor Microenvironment in isocitrate dehydrogenase Wild-type Glioma**

Jinwei Li^1#^, Shengrong Long^4, 5#^, Zhang Yang^7#^, Wei Wei^4, 5^, Shuangqi Yu^4, 5^, Quan Liu^6^, Xuhui Hui^3^, Xiang Li^4, 5*^, and Yinyan Wang^2, 3*^

^1^Department of Neurosurgery, West China Hospital, Sichuan University, Chengdu 610000, Sichuan, China

^2^Department of Neurosurgery, Beijing Tiantan Hospital, Capital Medical University, Beijing 100070, China

^3^Beijing Neurosurgical Institute, Capital Medical University, Beijing 100070, China

^4^Department of Neurosurgery, Zhongnan Hospital of Wuhan University, Wuhan, Hubei, China.

^5^Brain Research Center, Zhongnan Hospital of Wuhan University, Wuhan, Hubei, China.

^6^Department of Neurosurgery, The Fourth Affiliated Hospital of Guangxi Medical University, Liuzhou 545000, Guangxi, China

^7^Department of Vascular Surgery, Fuwai Yunnan Cardiovascular Hospital, Affiliated Cardiovascular Hospital of Kunming Medical University, Kunming, Yunnan, China

*Corresponding author

Xiang Li

[li.xiang@whu.edu.cn](mailto:li.xiang@whu.edu.cn) (https://orcid.org/0000-0002-6849-353X)

Yinyan Wang

[tiantanyinyan@126.com](mailto:tiantanyinyan@126.com) (https://orcid.org/0000-0003-1606-2284)

^#^These authors contributed equally: Jinwei Li, Shengrong Long, Zhang Yang


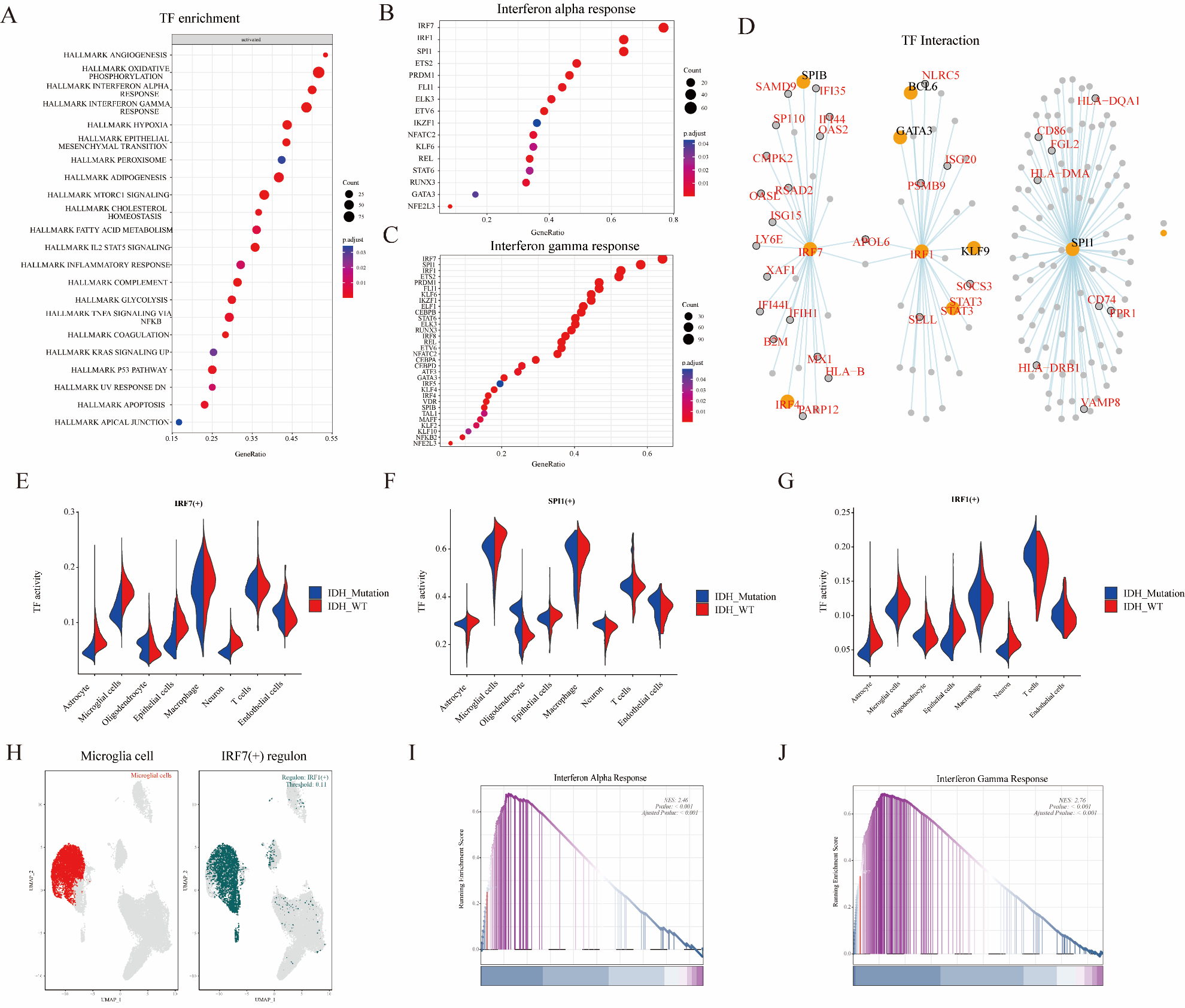


**Supplement Figure S1.** **Identification of key signaling pathways and genes in the gene regulatory network**. A. Scatter plot showed signaling pathways enriched for transcription factors. B. Scatter plot showed the percentage of transcription factors enriched in the interferon alpha response. C. Scatter plot showed the percentage of transcription factors enriched in the interferon gamma response. D. Interaction network diagrams show the interactions between IRF7, IRF1, and SPI1 transcription factors. E, F, G. Violin plots showed comparative expression of IRF7, SPI1, and IRF1 in IDH mutant and wild-type cell subpopulations. H. UMAP showed the distribution of expression in microglia and IRF7 regulon. I, J. GSEA showed the distribution of IRD7 in the interferon alpha response and interferon gamma response distribution.


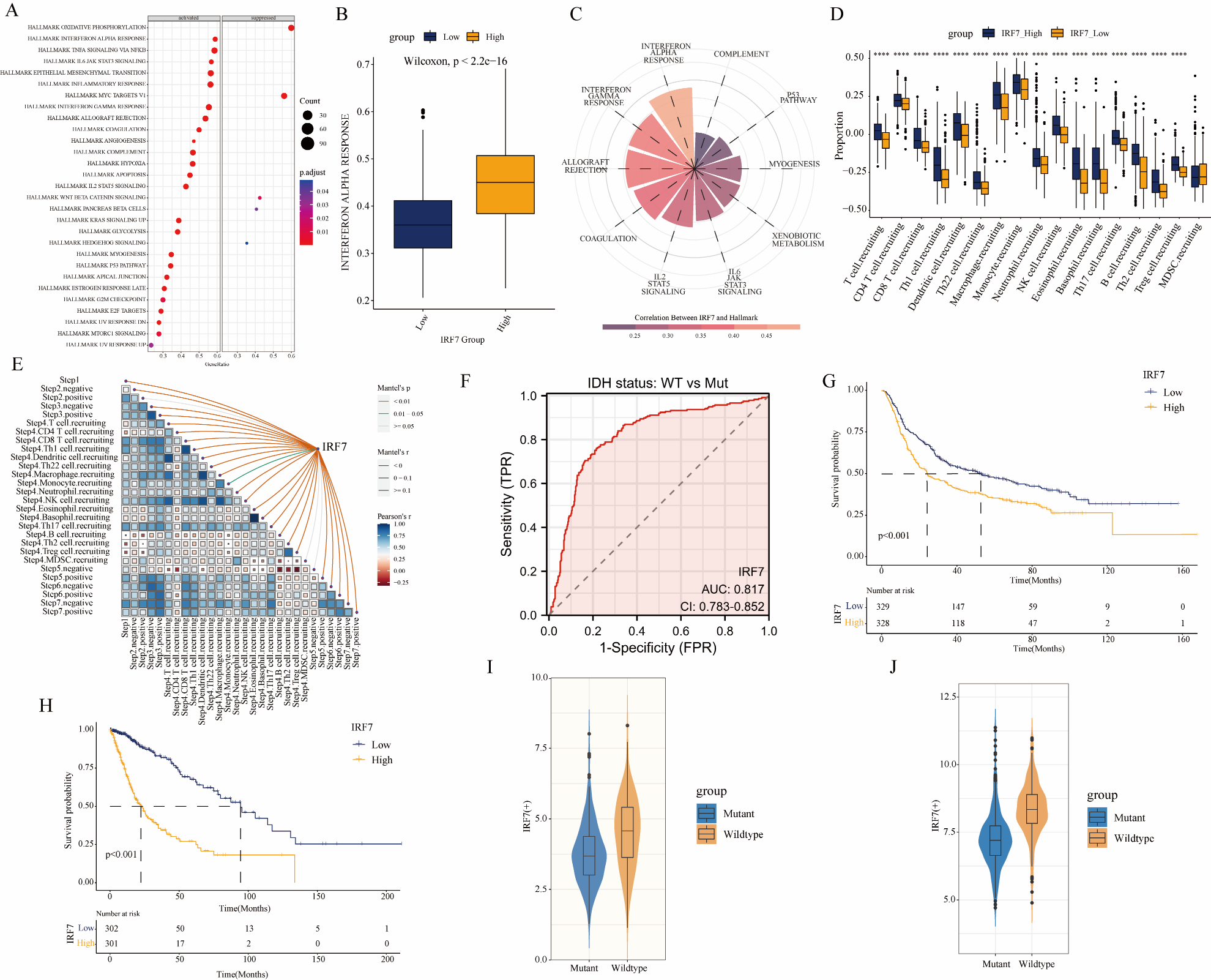


**Supplement Figure S2.** **The biological significance of the key gene IRF7 in transcriptome sequencing in glioma.** A. Scatter plot showed IDH mutation and GSEA signal pathway enrichment analysis of wild-type glioma differential genes. B. The box chart showed the Interferon alpha response scores of high and low IRF7 groups. C. The radar map showed the correlation between the IRF7 gene and the signal pathway. D. The box diagram showed the relationship between the high and low expression of the IRF7 gene and the infiltration of TIP immune cells. E. Heatmap showed the relationship between IRF7 and TIP immune cell infiltration. F. ROC curve showed the value of IRF7 in the diagnosis of IDH mutation and wild type. G. KM prognostic analysis showed the prognosis of high and low-risk IRF7 in TCGA and TIANTTAN-693 cohorts. I, J. The violin pictures show the expression of IRF7 in the TCGA and TIANTTAN-693 queues, respectively.


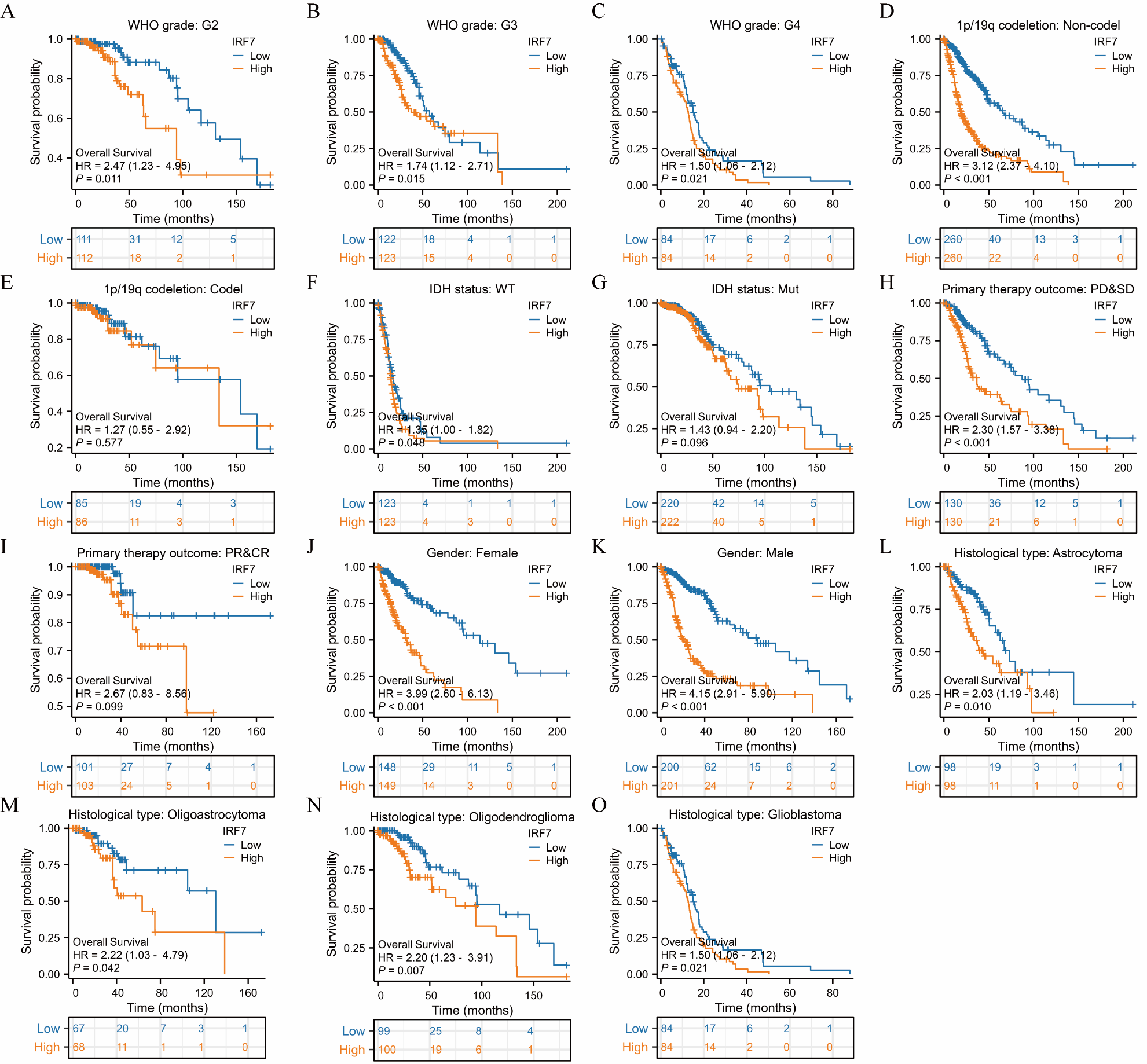


**Supplement Figure S3.** P**rognosis of IRF7 in clinical subgroups.** The prognosis of IRF7 in the clinical subgroups characterized by WHO II (A), WHO III (B), WHO IV (C), 1p19q non-co-deletion (D), 1p19q co-deletion (E), IDH wild-type (F), IDH mutant (G), PD&SD (H), PR&CR (I), gender (J, K), Astrocytoma (L), Oligoastrocytoma (M), Oligodendroglioma (N), and Glioblastoma (O).


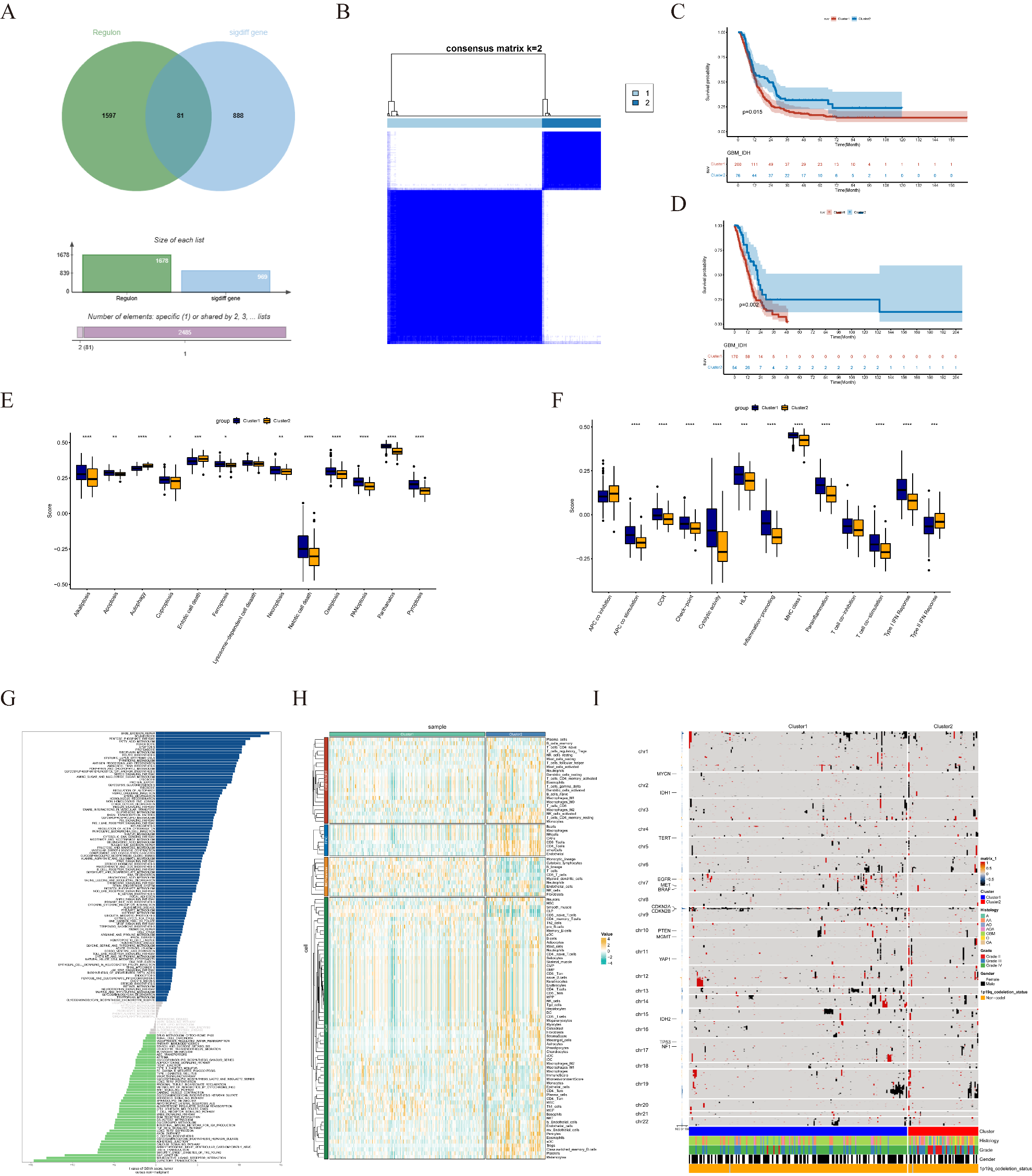


**Supplement Figure S4. Identification of IDH wild-type clinical subtypes.** A. Wayne diagram showed the subtype screening gene set. B. The heatmap showed the consistency clustering results. C. KM showed the prognostic profile of the C1 and C2 subtypes in the TIANTAN-693 cohort and the TCGA cohort. D, E. The box line plot showed cell death and immune function scores of the C1 and C2 subpopulations. F. GSVA enrichment analysis of up-and down-regulated signaling pathways in C1 and C2 subpopulations. G. Heatmap showed immune cell infiltration in C1 and C2. H. Heatmap showed Copy number alteration scores in C1 and C2 subpopulations. **p* < 0.05, ***p* < 0.01, ****p* < 0.001


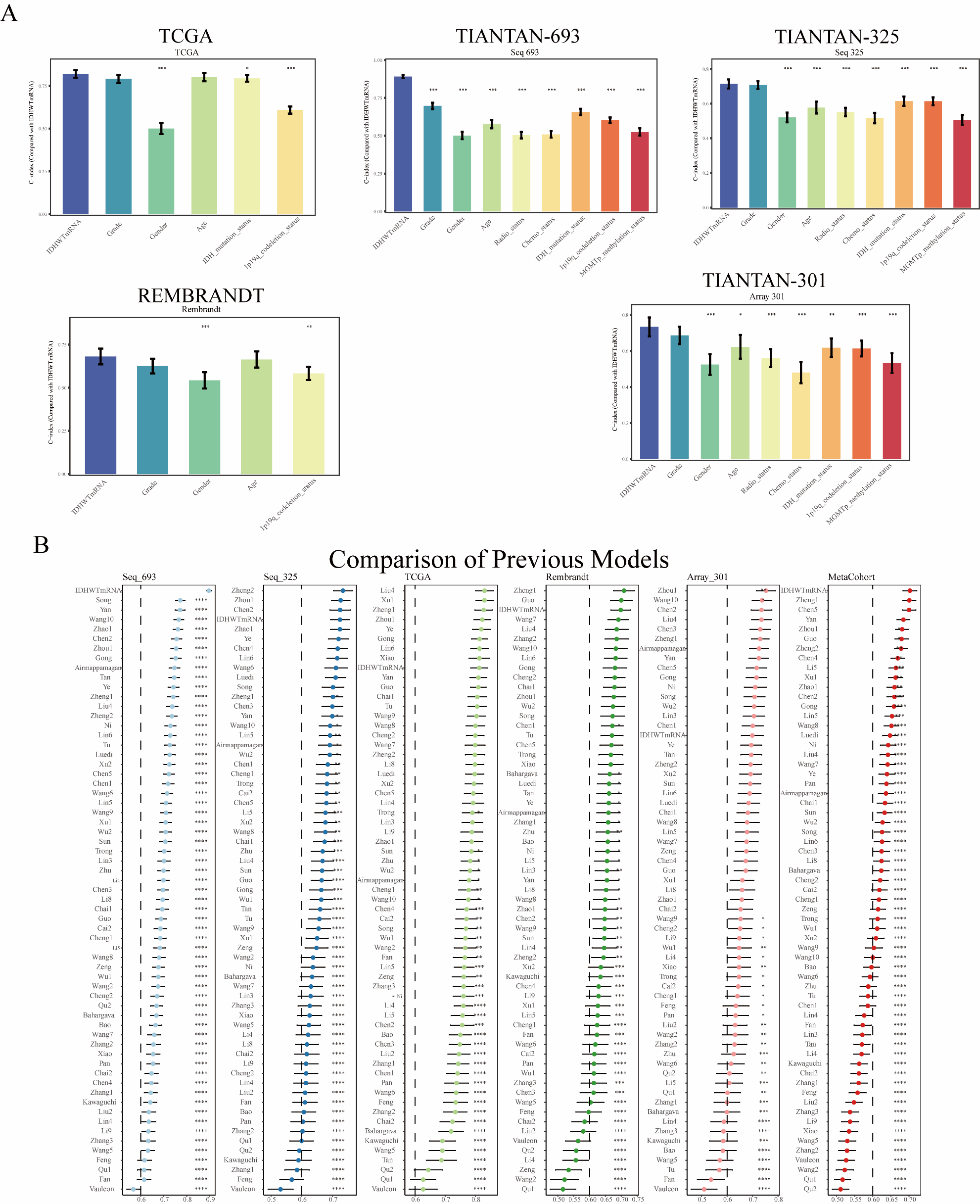


**Supplement Figure S5.** **Evaluation of the prediction ability of the model.** A. The box chart showed the clinical features compared with the model consistency index in multiple cohorts. B. Forest plot showed the comparative analysis of AUC values of the model with multiple previously published models. **p* < 0.05, ***p* < 0.01, ****p* < 0.001


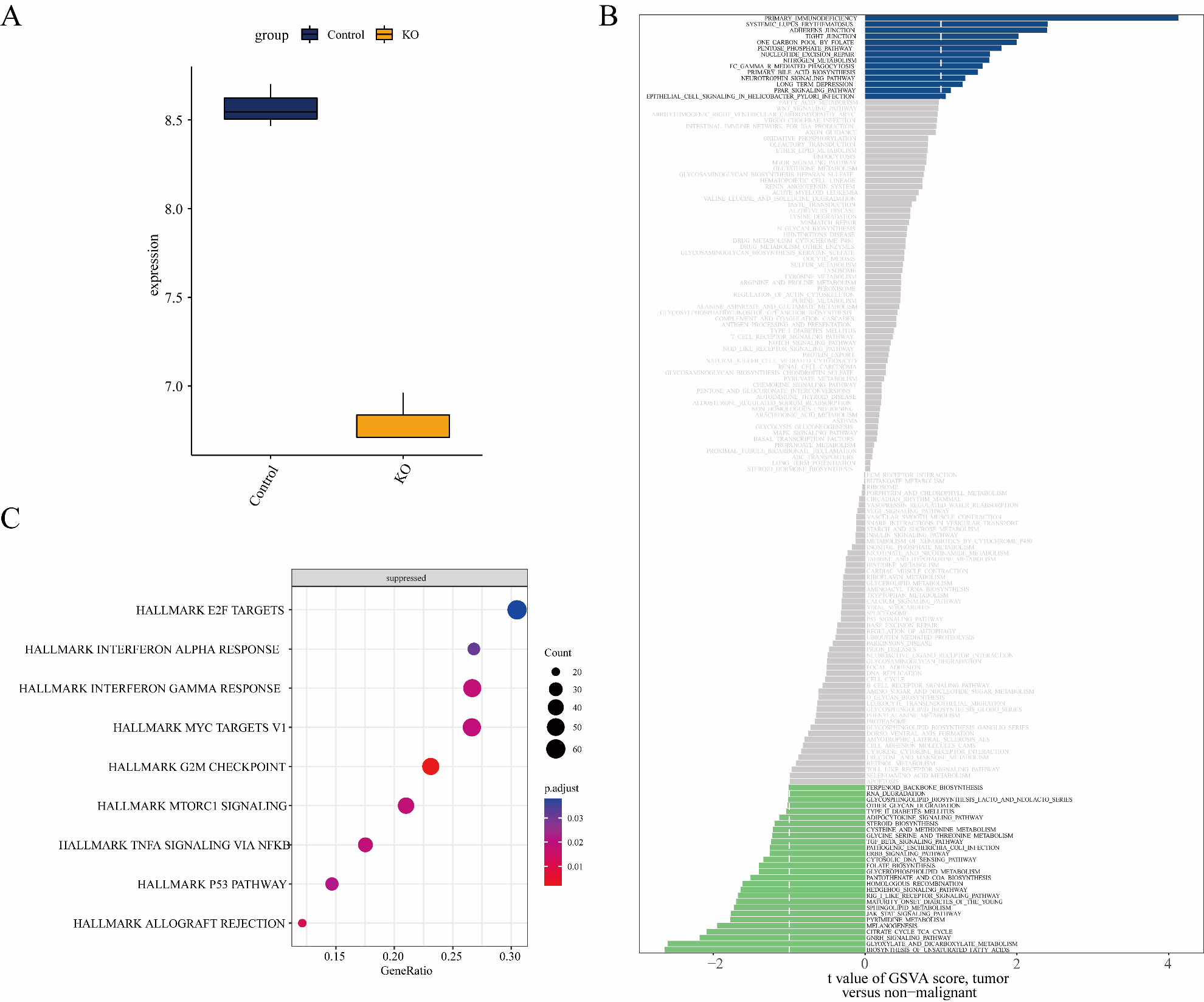
 **Supplement Figure S6. Transcriptome sequencing analysis of cell lines with knockout of IRF7 gene.** A. Comparison of IRF7 expression in U251-IRF7-siRNA and NC. B. GSVA enrichment showed up- and down-regulated signaling pathways. C. Scatterplot showed signaling pathways inhibited in GSEA enrichment analysis.

**Supplement Table 1. IRF7 Regulon**

| **IRF7 Regulon** | | | | | |
| --- | --- | --- | --- | --- | --- |
| IRF4 | UBE2J1 | MAPKAPK3 | INPP5F | ANO9 | CAST |
| MX1 | BTN2A1 | SPAG4 | TMEM214 | CCT6A | GALNT10 |
| HLA-F | SH3BP2 | ACSF2 | QSOX1 | CLEC2B | S100A13 |
| CMPK2 | ANKRD13D | LCP1 | AFTPH | PIM2 | BRWD3 |
| IFI35 | ARHGAP31 | CEACAM3 | TMCO4 | CLEC4A | UBE2B |
| LY6E | CD69 | TRAF3 | SULT1B1 | RAB35 | PDXDC1 |
| IFI44L | ZDHHC24 | BLVRB | SPN | CDC42EP3 | IVD |
| FUT7 | MECOM | CXCL9 | SLA2 | NECAP2 | EML4 |
| RSAD2 | RAMP3 | SLFN11 | TRERF1 | RGS16 | IFT57 |
| XAF1 | ERICH1 | CTBS | TSPOAP1 | LIF | F8 |
| IFIH1 | RBM38 | HDAC7 | PRKAG2 | ACSL4 | RECK |
| JCHAIN | SMPD2 | PCBD2 | ACAP2 | CXCR4 | AKAP13 |
| HLA-B | SLC25A19 | FFAR2 | LITAF | MELTF | STX18 |
| MZB1 | RRP12 | PYGL | CAV2 | RUSC1 | ZNF641 |
| LRRC26 | OLFML2A | GNG7 | HOXA1 | NLN | MFSD14B |
| OAS2 | HAPLN3 | NEK11 | SHBG | ERLIN1 | DYRK1A |
| PACSIN1 | SPINK2 | CTDP1 | CNEP1R1 | MDFIC | FKBP5 |
| IFI44 | TEP1 | SDC3 | EIF2AK3 | CXorf38 | SLC26A8 |
| APOL6 | FAM43A | TWF2 | JAML | RASGRP2 | TECPR1 |
| SMPD3 | PAK1 | CEACAM4 | TLR2 | TRPM4 | NRBF2 |
| B2M | BMPR2 | CPPED1 | PPM1M | PADI4 | LEMD2 |
| SAMD9 | LY6K | POMC | ZDHHC3 | STK10 | P2RY2 |
| ISG15 | TMEM102 | DENND1A | BATF3 | LILRB2 | KLHL5 |
| DERL3 | TMEM53 | NQO2 | AHR | CALHM2 | COMMD9 |
| OASL | TPST1 | VDR | RFTN1 | BST1 | EEF1D |
| GZMB | DGKA | TMOD1 | C20orf96 | MED26 | MAP3K1 |
| PARP12 | NUDT16 | KCTD9 | RASSF3 | PATL2 | TAOK3 |
| SPIB | GPATCH2L | MAP2K3 | MICALL2 | NEURL1 | SNRPB |
| SP110 | BFSP2 | AP1S2 | CSTB | EPS8L2 | CD81 |
| OAS1 | DDX3Y | SERPINA1 | IMPDH1 | ETV6 | PCK2 |
| TNFRSF17 | ULBP2 | TBX19 | GPR75 | GBP5 | SLC30A3 |
| PLSCR1 | WWP1 | IL15 | SNAI1 | EVA1C | LSMEM1 |
| DTX3L | HOMER3 | C9orf64 | ERGIC1 | PRPH | LILRB3 |
| GBP4 | HAUS2 | ISG20 | PACS1 | TLN1 | FTCDNL1 |
| MX2 | SSR4 | RUNX3 | NOD1 | SIRPB1 | PLS1 |
| CLIC3 | SDE2 | ZNF629 | MMP23B | CD72 | DISC1 |
| IFIT3 | FBXO38 | PTPN2 | P2RX1 | KIAA0040 | FAM114A1 |
| TCL1A | ZFP36 | CKLF | MBOAT7 | PTPRCAP | DPAGT1 |
| MYD88 | MKNK2 | CD79B | PSTPIP2 | GSDMD | MTM1 |
| DDX60L | ARHGAP27 | CES3 | LRP10 | ITPKB | RASSF8 |
| DDX58 | EXOSC3 | ANTXR2 | RHOA | VSIG10L | PARP3 |
| ZC3HAV1 | PCED1A | MPZ | UFSP1 | DEFB1 | CD3E |
| DDX60 | PHKA2 | ERO1A | VSTM1 | CCR7 | ACOX2 |
| RTP4 | PHRF1 | STXBP2 | GPR183 | RAB11FIP1 | CTSK |
| IFI16 | TMEM248 | FCRL5 | RAC2 | RUFY1 | MT1E |
| SLC2A3 | CAPN15 | JSRP1 | SULT1A1 | ALDH3A1 | ADAMDEC1 |
| SAMD9L | SH2D1B | MMP14 | SLC35F3 | TRIM22 | MYO5C |
| PARP9 | NOTCH4 | HLA-DOB | GOLPH3 |  |  |
| HERC6 | IL18RAP | KLHL26 | TAP1 | KLHDC8B | NR4A2 |
| HDHD3 | CHPF2 | DNAJC4 | CTDSP1 | FSBP | SERPINB1 |
| ZFAT | FCHO2 | PPARD | GLB1 | ICOSLG | NAPA |
| KLF10 | LIMD1 | HLA-DPA1 | YBX3 | SKAP1 | HDAC9 |
| IFITM3 | TRANK1 | IL27RA | BHLHE40 | ARFGAP3 | APOBEC3C |
| NME8 | LYN | DNAJC13 | BTN3A2 | FFAR3 | NOL6 |
| STAP1 | KCNJ5 | MAFF | LAMP3 | PPP3R1 | RILP |
| MSN | MYL12B | RORC | ABCA7 | GATA3 | PDK1 |
| MALT1 | LXN | CACNA2D3 | MOB3A | TPMT | RPS6KB2 |
| CRYM | PIP4K2C | SMPDL3A | OPN3 | COQ7 | SKI |
| SH3BGRL3 | AGTRAP | TEKT3 | POU2AF1 | AP4E1 | CHML |
| BCL3 | KLF4 | ETV5 | PTPN1 | KCNF1 | CFI |
| ATP1B3 | ANKRD42 | RXRA | FLT3 | FBXL19 | CCDC57 |
| NMI | SZT2 | ID2 | SLC25A43 | GATM | FES |
| TMEM127 | MAP4 | CASP1 | RAP1B | PDE6G | SLC9A7 |
| FABP5 | PANX1 | GPR65 | SLC12A3 | MOB1A | CD200R1 |
| PTCRA | HOXB2 | CIB1 | ICAM1 | GGA2 | CRTAM |
| AMN | TRPS1 | SERPINB8 | ERAP2 | STK19 | FCER1A |
| DHX58 | CASP3 | VRK2 | RHOQ | GRK5 | POLD1 |
| IFI6 | CSRP2 | RELL2 | PDXK | SMCHD1 | CEP135 |
| BCL11A | LPCAT3 | PLEKHM2 | NFE2L3 | LRG1 | MLF1 |
| PSMB9 | ZNRF2 | MICB | NFKB2 | HEBP1 | PPP1R15B |
| UBE2L6 | ATAD2B | ARL4A | SELL | ZCCHC2 | MCEMP1 |
| NFIL3 | NUDT3 | SLC34A2 | RNF149 | SIRPG | MR1 |
| IFITM2 | EHBP1L1 | CPNE7 | GNPTAB | ETS2 | CCL13 |
| TTC39A | VCX | GPR132 | PRSS12 | ZNF215 | PSMB10 |
| TRIM21 | CAMK1 | NCF1 | ELL | ARRDC1 | EFHC2 |
| ASL | RAB11FIP5 | HSPB1 | SIK1 | PNRC1 | PCGF5 |
| P2RY14 | CDC14A | SLC7A5 | LAD1 | UBC | TDRD7 |
| MAFK | ARCN1 | STEAP3 | IL1R1 | COX17 | PIK3CG |
| GBP3 | HERPUD1 | MYCBP | SPTY2D1 | JMJD1C | CDV3 |
| STAT1 | BICDL2 | CD8A | LTK | CDK2AP2 | ZNF277 |
| OAS3 | FRMD3 | FOXP3 | PNOC | FBXW5 | SEMA4A |
| TAPBP | DAZAP2 | GLRX2 | ANGPTL1 | GLE1 | TAF12 |
| IFIT1 | KLHL15 | OPA3 | AREG | PPP1R18 | ULK4 |
| LILRA4 | MEPCE | PHF11 | UTRN | PPTC7 | HOXB6 |
| ERAP1 | ZNF124 | DALRD3 | GRIP1 | MED30 | MAP1LC3A |
| TNFRSF14 | CMTR1 | IL2RG | STAT6 | SLC25A32 | C2CD4A |
| CXCL10 | CPA6 | NUP62CL | MCTP1 | TFE3 | TMEM159 |
| PHEX | TRABD2A | CPNE8 | B3GNTL1 | TTC32 | PF4 |
| RELB | OR51A7 | LAG3 | PTK2B | KIF3C | BCAM |
| GCH1 | VRK1 | TMEM176B | YPEL5 | ACSM3 | LIPN |
| INPP4A | NR4A1 | GLRX | PTEN | GMPR | PRAM1 |
| ANXA1 | SEC14L3 | GPA33 | ERN1 | SPRYD3 | FAM20A |
| TRIM14 | APOBR | ANKRD29 | MAPKAPK2 | CHD1 | TMEM150A |
| EPSTI1 | CLIP1 | SCLT1 | FURIN | ZNF468 | EID3 |
| FAM221B | TEX14 | FCRL2 | FBXO6 | HGF | CALCB |
| VMP1 | CD70 | TP53INP2 | TMSB10 | MBNL3 | ARF6 |
| CCR2 | BCL2 | DNAJC5 | SLC25A30 | APOBEC3F | WDSUB1 |
| GBP2 | ECHDC2 | MAD1L1 | TRIM25 | ADGRG3 | SWAP70 |
| NFKB1 | TMEM229B | STK26 | GADD45B | MYADM | KIF16B |
| CLIC1 | TESMIN | COTL1 | TES | FAR2 | OMA1 |
| SOCS3 | MYO5B | HOXB4 | SEMA7A | RNF43 | APTX |
| MROH1 | ZNF366 | CLEC4F | GSTK1 | COQ10B | GTF3A |
| GSX2 | ADAM8 | USP32 | TICAM1 | EAF2 | SYNE3 |
| PML | CROT | PIEZO1 | SLC15A4 | ZNF331 | P4HA2 |
| CRIP1 | UBAP1 | HMGCL | DPEP2 | VAMP4 | TAB2 |
| LGR6 | ARHGAP29 | ABCD1 | MYL12A | ATP6V1F | PDCD10 |
| GLT1D1 | UBA1 | DIAPH1 | ADAR | PUS10 | GK5 |
| HEXB | CCDC6 | RPP25 | APOL1 | LINC00514 | GYG1 |
| RNASEL | TNFAIP8 | SULT1C2 | MAP3K7CL | R3HDM4 | CCSER1 |
| MRPS17 | MFSD13A | VCL | NEDD9 | IRF2 | CUL7 |
| HLA-A | PBX3 | VSIG2 | SDCBP | HS3ST4 | ABRACL |
| FAM160A1 | ITPR3 | CYB5D1 | VILL | DEDD | PRELID2 |
| TPM3 | GPR157 | PFN1 | C9orf72 | RAP2B | RCAN1 |
| HELZ2 | PRKAR2B | HLA-DPB1 | SP100 | SNRK | ADAMTS3 |
| CXCL11 | SFTA2 | MRPS6 | EPS8L1 | RPN1 | NOD2 |
| TPM2 | LYSMD3 | ACTR3 | ZDHHC7 | AP2A2 | NTRK1 |
| HERC5 | ALAS1 | CNPY4 | ZNF217 | N4BP2L1 | CD58 |
| SOD2 | OTULIN | ZFP57 | C15orf39 | SPATA2L | PUS1 |
| NAMPT | HGSNAT | POR | SLC25A37 | SLC6A16 | KMO |
| ZBTB46 | CYYR1 | RIPK1 | NAPRT | E4F1 | PLXNC1 |
| IRF1 | TEX264 | PAX5 | SCT | PPFIA1 | ZNF267 |
| CXCR3 | PSTPIP1 | SLC30A7 | EZR | SMIM12 | ASAP3 |
| FHL3 | OGFR | MYH9 | ACOT9 | LRRC25 | DNAJC5B |
| PARP14 | KCTD20 | CNN2 | TTC38 | EPAS1 | RALGAPA2 |
| FOSL2 | TAF13 | TRIM47 | CD300E | KIAA0753 | ARL5B |
| RNF19B | PLA2G2C | SLC19A1 | MTHFS | TESPA1 | JAK1 |
| AMIGO2 | CCDC71L | CLEC4E | PODXL | HECTD2 | CATSPERB |
| KCNA5 | BIRC3 | CSRNP1 | FMNL1 | PLAUR | ROCK1 |
| HPCAL1 | HHIPL1 | HSPA1A | SPSB2 | ACOT4 | SPG11 |
| PARP10 | NPHP4 | TMEM107 | SERP1 | MOCOS | IRF8 |
| ETV3 | HLA-DOA | FKBP1B | IL17RC | CSTA | OR52K2 |
| PLAC8 | BICD2 | G6PD | ATP2B1 | PIGZ | RUFY4 |
| RAB8A | MAOB | FLOT1 | CD55 | CAT | TMEM63B |
| CD59 | SLC7A11 | GNA15 | TSPAN32 | TMEM255B | GALNT3 |
| RAP1GAP2 | PCYOX1L | HEG1 | NIPSNAP3A | PANK2 | ATAD3C |
| SHISA5 | PLAGL2 | JUNB | PPIC | CNOT6L | FAM83G |
| PSMB8 | EPHX3 | SYCP2L | NAAA | SLC25A20 | ADTRP |
| DUSP5 | JOSD1 | STK38 | DNAJA1 | ARL13B | CDH23 |
| RUBCN | CLN8 | GLTPD2 | ESR2 | COPB1 | ZNF888 |
| DNASE1L3 | MAPK1IP1L | CYLD | MVP | CXCR5 | CHFR |
| PDE4A | MORN1 | SH3GLB1 | CRTAP | KRT36 | CD209 |
| GBP1 | TNFSF13 | ADAMTS15 | MOV10 | ADAM17 | AAGAB |
| KRT5 | C1R | GPCPD1 | KCNK17 | YWHAB | FAM181A |
| CCL22 | PRSS16 | TNFAIP3 | STARD4 | ITGB4 | SLC35A2 |
| LRRFIP1 | AIFM3 | CREM | TNIP1 | EMP2 | SPPL2A |
| IRAK1 | CFP | CASZ1 | VMO1 | EIF4EBP2 | CHKB |
| RRAS | SF3B4 | DUSP4 | BTN3A1 | NLRP12 | SLC2A4RG |
| PPP1R2 | IL27 | ZNF214 | MSL3 | SLC22A4 | MYO15A |
| LACTB | CALCOCO2 | LIMK2 | CCR3 | HEATR3 | TMEM39A |
| STK17B | FAM24B | PRICKLE3 | CBFA2T3 | FAM78B | IQGAP2 |
| UPP1 | RHOBTB2 | HLA-DRA | NRP2 | RSPH9 | TBC1D16 |
| BTN3A3 | FHAD1 | TRIP10 | OXT | TMEM222 | ABRA |
| ARPC3 | PTPN7 | DENND1B | SLC25A24 | APRT | FTH1 |
| SERPING1 | WDFY2 | EVC2 | CMTM3 | STK3 | IDH3A |
| EDEM2 | CYSLTR1 | PLB1 | ETF1 | KCNMA1 | ZNF672 |
| CCDC69 | SCPEP1 | SEMA3E | IL11RA | ARFRP1 | CORO2A |
| SIL1 | EHD4 | IRAK4 | USP18 | BANP | LGALS8 |
| STAT4 | CYP2A6 | IL4R | KLHDC7B | SERGEF | IL1RN |
| ARPC4 | LATS2 | ADGRE2 | AP1S3 | EML3 | ZCCHC10 |
| ACSL5 | CFAP61 | ZFP36L2 | PRMT9 | PTGR1 | LCP2 |
| VASP | NXN | DNASE2B | ZFAND5 | PIK3R5 | GMPPA |
| DRD4 | SMU1 | MSL2 | PPM1J | TRAF7 | CYB561A3 |
| TMEM176A | PTGDR | CD79A | KIAA0513 | DSE | PERP |
| GNS | TAX1BP3 | CCDC186 | SNX20 | TNIP2 | SNX9 |
| COL24A1 | LRRC43 | APOBEC3A | HES4 | TBC1D23 | GDPD5 |
| TRIM69 | LGALS3 | NUAK2 | CSF2RB | NUDT6 | ARRDC4 |
| BTG1 | YWHAZ | DUSP10 | PPCS | OXSR1 | NIPBL |
| RAB27A | EIF4E3 | WHAMM | BRI3 | MTMR14 | APOBEC3G |
| PLTP | ZFAND1 | ARID3B | RHOH | NFKBIB | PITPNA |
| GAPT | P2RY10 | ATF3 | SIT1 | VAV1 | EDEM1 |
| PIM1 | ZFAND4 | PARP11 | STK38L | SOAT1 | PLIN5 |
| MCOLN2 | TXNRD3 | AHNAK | CHMP1B | IFFO2 | ALG6 |
| MAP3K8 | TSPAN17 | ABHD5 | NECTIN2 | EIF2AK4 | PARM1 |
| PITPNM1 | ARNTL | LYST | TLE3 | FAM117B | DAP |
| KLF11 | SLC16A1 | MPZL3 | BCL2L11 | HTRA1 | GZMM |
| TIPARP | SPDYA | CXCL2 | ENSA | CD6 | CD300A |
| SBNO2 | MIER1 | SOCS1 | ODF3B | MYLPF | S1PR4 |
| USP36 | IQCA1 | IL7 | TBC1D8 | CD300C | IL1R2 |
| S100A10 | PKP2 | SP3 | RBCK1 | SPAG1 | DDX21 |
| ARPC2 | ATG5 | SCMH1 | CCDC183 | ACSL1 | MPEG1 |
| BAK1 | BACH1 | PROS1 | BANK1 | TNFSF15 | RCOR1 |
| NLRC5 | DHX37 | LTF | IL10RB | C22orf42 | SYT12 |
| RAB29 | CCDC107 | PFKFB2 | SAMHD1 | WASL | HMGA1 |
| TNFRSF9 | ITGA6 | PRKD2 | DCAF6 | ICOS | S100A6 |
| AGPAT2 | MYCBPAP | TPRG1L | TAP2 | KCNAB2 | DTNBP1 |
| RIPK2 | C16orf72 | MPZL2 | SEL1L3 | NOTCH2 | MEFV |
| F11R | SNTA1 | PLEKHG2 | ARID5A | CADPS2 | ITPKC |
| PSME2 | FAM20C | USB1 | DUSP2 | MAPK7 | AMDHD1 |
| PLEC | IFNL1 | EPHB4 | RNF213 | WDR55 | SLC12A7 |
| EIF2AK2 | ATG16L2 | GRAMD4 | RGS2 | HAS1 | E2F4 |
| TAPBPL | ACP5 | SLC19A2 | PSME1 | PHC3 | FOSL1 |
| TFRC | MGAT1 | ZNF410 | SLC20A1 | NACA | SIGIRR |
| FXYD5 | STYX | TTC7A | DNM1 | EDA | NR1H2 |
| GLIPR2 | TNFRSF1B | TMEM61 | C5orf49 | DENND4A | CD19 |
| CASP4 | CCDC22 | ARHGEF35 | PNP | RYBP | PHTF1 |
| ST6GALNAC4 | GNGT2 | ACAP1 | NBEAL2 | ADGRG5 | CBLN3 |
| ZNFX1 | PDCD1 | ABHD15 | OAZ1 | PLXNA3 | CD244 |
| TMEM71 | CHST13 | SLC44A3 | INSIG1 | MCTS1 | MAN1C1 |
| BTLA | SIGLEC1 | UTF1 | SH2D3C | CAPNS1 | MS4A1 |
| HSD3B7 | SEC61A1 | SFTPD | LASP1 | CAPZB | NUMBL |
| NDEL1 | IVNS1ABP | LPCAT1 | TNFRSF1A | PLEKHF2 | GPR18 |
| CAPN1 | TMBIM1 | APOBEC3H | CYB5R1 | YDJC | NUB1 |
| XYLB | RHOF | IQSEC1 | RNASEK-C17orf49 | PRR15 | ZNF622 |
| ICAM3 | IL18R1 | SLC25A25 | AIM2 | NFE2L1 | TMPO |
| RPS6KA3 | BCL10 | PVR | SLC39A1 | PDE7A | WDR45 |
| LDHA | TBC1D1 | FAM3B | PLIN3 | ACKR3 | BASP1 |
| SIDT1 | PLD2 | ZFAND6 | SLC9A1 | ATG7 | KDM6B |
| PRDM1 | PTGIS | CLK3 | MIA | GPR84 | TXNL4B |
| BCL2A1 | N4BP1 | C11orf24 | UBXN11 | LRRC2 | C1RL |
| SCN9A | ZNF264 | DNAJC14 | DERL1 | ENY2 | ZNF699 |
| HSH2D | NADSYN1 | ANKMY2 | CCND3 | PNPLA2 | KYNU |
| NCR3 | EGR4 | HACD4 | POLB | NIT2 | FAM117A |
| ATP1A1 | ASB8 | ARF1 | RAB3D | PTTG1IP | NEFH |
| G0S2 | ATG2A | CMTM6 | HESX1 | JAK3 | ATP6V1B2 |
| TRIM56 | CTDSP2 | WNT5A | IL13RA2 | MCCC1 | ZNF33B |
| ST20 | DNAAF1 | P2RX5 | EMP3 | EFR3A | ATP8B1 |
| S1PR3 | POPDC2 | HES1 | BAZ1A | EFHD2 | ABCC6 |
| STARD5 | LPO | FAM120AOS | NSMAF | DVL3 | MACC1 |
| RILPL2 | MMP8 | GCA | CLEC10A | SETD1B | FRMD4A |
| SP140L | ITIH3 | TTC23 | SNPH | THBS1 | PPP1R12A |
| NR4A3 | CLEC4G | IDO2 | RNPEPL1 | MYL5 | ZNF782 |
| BTN2A2 | PSMA8 | NPIPB11 | HUS1 | RIC1 | NRIP3 |
| ADGRE5 | LYPD2 | TMEM42 | HOXB3 | CTNS | GCSAM |
| CD1C | LINGO4 | C5AR2 | KCNG2 | TIFAB | MAPRE1 |
| RAB8B | DKK2 | FKBP1A | LILRA5 | CCDC159 | RELT |
| FAM214B | CNR2 | NPL | PDK3 | WDR26 | DUSP22 |
| CHIC2 | PLEKHG6 | SORL1 | CCDC9 | CD163L1 | MFSD11 |
| MBD2 | XDH | B3GNT2 | HLA-DQB1 | FAM118A | ATP11C |
| SERPINF2 | MBOAT4 | GPR82 | SNX10 | UTP23 | NHLRC4 |
| GGA3 | FCER2 | SPCS3 | CD48 | AQP3 | ADGRE1 |
| ZNF385A | GALNTL6 | NRROS | STIM1 | STAT3 | ZRSR2 |
| IL1RL2 | FCRL1 | TFB2M | ZBTB32 | PRPS2 | FAS |
| IRF7 | ASTL | ZNF28 | MLKL | JAZF1 | SEC24D |
| TXNDC11 | FCRL3 | RPAP3 | SUMO3 | SH3D21 | HBEGF |
| NEMP1 | CDH17 | FBXO25 | CD44 | RPA3 | CIITA |
| MYO1G | XCR1 | PCNX1 | LAMP5 | RAB31 | CMIP |
| UBE2D3 | FOXP1 | ATP9B | GALNS | SLC16A5 | SHKBP1 |
| BCAT1 | STK40 | MOGAT1 | ITGA4 | UPK3A | REL |
| PDP1 | PIGB | PKD2L2 | LY9 | RNF212 | ANXA11 |
| ASXL2 | ZBP1 | GLMP | EPHB6 | ZNF525 | TBC1D10C |
| MGARP | ARHGAP9 | AEBP1 | IGLL5 | FGGY | ANKRD37 |
| TPCN2 | LUZP1 | AOC1 | HMGB2 | SPOPL | ZNF394 |
| NADK | ZNF296 | HSPB11 | RNF144B | ZNF354A | MFNG |
| MYB | CITED2 | GLS | HTATIP2 | IDH1 | ARL5C |
| ITGA5 | ANO6 | GLA | AFAP1L1 | ZNF18 | GSR |
| IDNK | TESK1 | IDO1 | PCDHGA12 | SAP30 | RUNX2 |
| NFE2L2 | TNIP3 | CLDN1 | SDSL | SLC38A4 | PCP2 |
| ERP44 | HIC2 | CDH4 | COL8A1 | CGGBP1 | GPAT3 |
| UGCG | NCOA2 | MAP7D3 | HS3ST3B1 | ADAMTS17 | TGIF1 |
| ZDHHC12 | MFSD12 | CD1E | PTGER4 | SESN2 | NIPAL2 |
| CFLAR | ATXN7L3 | STK24 | APOL3 | PHYKPL | FOLR3 |
| SLC35C1 | CYP4F22 | TRAT1 | SPIDR | ACTN1 | TRIM5 |
| DYNLT3 | TMEM243 | HM13 | IFNAR2 | UBA7 | MAP1LC3B |
| ZNF600 | ARHGDIA | CD207 | ABCC1 | CTF1 | C12orf75 |
| SLC18A2 | PTX3 | C22orf15 | PTPRE | PPIF | AZIN1 |
| ZYX | CTC1 | SLA | ATP6V1C1 | ARHGEF3 | ABCB8 |
| PBX2 | MBNL1 | OSGIN1 | AP5B1 | APOL2 | SHQ1 |
| TPM4 | C7orf26 | CFB | TMEM234 | ST14 | UBALD2 |
| TANK | ASB6 | ABCA1 | TAGLN2 | DAPK1 | ADGRE3 |
| SECTM1 | RBM3 | HIVEP2 | HECA | HK2 | COBLL1 |
| HIVEP1 | CST3 | RMDN2 | TOMM40L | GSAP | OXGR1 |
| SLC50A1 | PDE4B | ANKH | FAM111A | DSG2 | SEC31A |
| PIM3 | GALNT6 | CACNA1F | C16orf74 | GPR155 | TOP1 |
| MBOAT1 | TFAP2C | SUSD1 | LTBP1 | TRPV2 | SQSTM1 |
| TET2 | FYTTD1 | PSMF1 | RALB | EHHADH | EXOC4 |
| TBK1 | EBAG9 | FYCO1 | COMTD1 | CD40LG | GABARAPL1 |
| PLA1A | TOR3A | SSC4D | CLIC2 | SLC37A1 | SCN4B |
| WNT10A | SIGLEC11 | DOCK6 | RIT1 | IL1B | UEVLD |
| TNFRSF13B | TMEM69 | S100A11 | S100A16 | VPS37C | S100A12 |
| ASB2 | TXLNB | ADIPOR1 | YIPF1 | THEM6 | PHLDA2 |
| SLC6A6 | LYVE1 | ETV7 | PATJ | IQSEC3 | FGFRL1 |
| ICAM4 | CHSY1 | MAP2K1 | IFIT2 | ECM2 | CTNNB1 |
| DPY19L1 | FBXO33 | CCDC8 | NFATC1 | ZDHHC5 | MTCP1 |
| CCRL2 | B4GALT4 | ULBP1 | ASIP | CTNNBL1 | PPP2R5A |
| LTB | BLK | ZFAND2A | PLP2 | ANXA2 | MICAL2 |
| SLC22A18 | KRT8 | TIGAR | TSPAN4 | MEI1 | SLC35F5 |
| DBNL | AK8 | DNPEP | PTBP3 | FOXJ1 | CCNYL1 |
| WIPF1 | NEMP2 | TRAM2 | SYS1 | ZG16B | UBXN10 |
| SLAMF7 | LAIR2 | RNPEP | ADARB1 | RHEBL1 | SRGN |
| STX3 | SLC27A4 | VWA5A | AMPD2 | CNGA3 | CSK |

| **Subtype typing gene sets** | | |
| --- | --- | --- |
| HLA-F | EHBP1L1 | ZYX |
| HLA-B | PSTPIP1 | ASIP |
| GBP2 | OGFR | TSPAN4 |
| HLA-A | C1R | PRPH |
| TPM2 | SEC61A1 | PTPRCAP |
| PARP10 | SPATA2L | GSDMD |
| PSMB8 | ITGB4 | MICALL2 |
| SIL1 | EML3 | MMP23B |
| DRD4 | TRAF7 | IRF7 |
| SBNO2 | TNIP2 | ACSF2 |
| NLRC5 | MAPK7 | MMP14 |
| ABCA7 | PLXNA3 | MAD1L1 |
| B3GNTL1 | DVL3 | PIEZO1 |
| EPS8L1 | UBA7 | ABCD1 |
| MOV10 | ZNF296 | POR |
| VMO1 | DOCK6 | TRIM47 |
| HES4 | AK8 | LTF |
| TLE3 | CTNNBL1 | PRKD2 |
| ODF3B | FOXJ1 | PLEKHG2 |
| MIA | DNAAF1 | CCDC57 |
| HOXB3 | AKAP13 | POLD1 |
| CD44 | EEF1D | BCAM |
| SDSL | AEBP1 | FAM20A |
| COMTD1 | HM13 | PUS1 |
| CAPN15 | SH3BP2 | SLC2A4RG |
| HOXB2 | HAPLN3 | GMPPA |
| SHKBP1 | PCED1A | UBALD2 |

**Supplement Table 2. Subtype typing gene sets**

**Supplement Table 3. Prognostic model gene sets**

| **Prognostic model gene sets** | | |
| --- | --- | --- |
| ABCD1 | LTF | GBP2 |
| AEBP1 | MIA | GSDMD |
| AK8 | MICALL2 | HES4 |
| B3GNTL1 | NLRC5 | HLA-A |
| C1R | ODF3B | HLA-B |
| CAPN15 | PARP10 | HLA-F |
| CD44 | PCED1A | HOXB2 |
| COMTD1 | PLXNA3 | HOXB3 |
| DNAAF1 | POLD1 | IRF7 |
| DRD4 | PRKD2 | ITGB4 |
| EEF1D | PSMB8 | SBNO2 |
| EPS8L1 | PSTPIP1 | SDSL |
| FAM20A | PTPRCAP | SEC61A1 |
| FOXJ1 | TNIP2 | SHKBP1 |
| UBALD2 | TSPAN4 | SLC2A4RG |
| ZYX |  |  |

**Supplement Table 4. The 65 collected signatures**

| **Model** | **Journal** | **PMID** | **Cancer** | **Author** |
| --- | --- | --- | --- | --- |
| Model1 | Biomark Insights | 21234290 | GBM | Zhou1 |
| Model2 | BMC Med Genomics | 22980038 | GBM | Vauleon |
| Model3 | PLoS One | 23646114 | GBM | Airmappamagan |
| Model4 | CNS Neurosci Ther | 23663361 | GBM | Bao |
| Model5 | Cancer Sci | 23745793 | GBM | Kawaguchi |
| Model6 | Neurology | 27225222 | GBM | Cheng1 |
| Model7 | BMC Syst Biol | 27586240 | Glioma | Wu1 |
| Model8 | J Neurosurg Anesthesiol | 27653222 | GBM | Luedi |
| Model9 | Oncotarget | 27713134 | GBM | Wang1 |
| Model10 | Mol Neurobiol | 27900679 | GBM | Zhao1 |
| Model11 | Oncotarget | 28035070 | GBM | Bahargava |
| Model12 | J Genet Genomics | 29169920 | Glioma | Chen1 |
| Model13 | J Cancer Res Clin Oncol | 29299749 | GBM | Chai1 |
| Model14 | Zhong Nan Da Xue Xue Bao Yi Xue Ban | 29774872 | GBM | Chen2 |
| Model15 | Int J Mol Sci | 30257451 | Glioma | Trong |
| Model16 | Theranostics | 30279734 | GBM | Wang2 |
| Model17 | Aging (Albany NY) | 30810537 | Glioma | Chai2 |
| Model18 | Biomed Res Int | 31032367 | GBM | Hou |
| Model19 | J Transl Med | 31097021 | Glioma | Sun |
| Model20 | Epigenomics | 31272213 | LGG | Li4 |
| Model21 | Oncol Lett | 31289499 | Glioma | Yan |
| Model22 | Oncoimmunology | 31428519 | GBM | Zhu |
| Model23 | J Cell Mol Med | 31475440 | LGG | Wu2 |
| Model24 | Front Genet | 31632439 | GBM | Cheng2 |
| Model25 | J Cancer | 31632497 | GBM | Ye |
| Model26 | Aging (Albany NY) | 31844032 | GBM | Wang5 |
| Model27 | J Neurooncol | 31853837 | LGG | Song |
| Model28 | Cell Commun Signal | 31907037 | Glioma | Zeng |
| Model29 | J Cell Biochem | 31960992 | GBM | Pan |
| Model30 | Onco Targets Ther | 32021258 | GBM | Wang6 |
| Model31 | J Cancer Res Clin Oncol | 32060643 | Glioma | Liu2 |
| Model32 | J Cell Mol Med | 32065482 | GBM | Wang7 |
| Model33 | J Cell Mol Med | 32160398 | LGG | Li5 |
| Model34 | Ann Transl Med | 32309356 | LGG | Ni |
| Model35 | Front Genet | 32351547 | LGG | Zhang1 |
| Model36 | Cell Mol Neurobiol | 32410107 | GBM | Xia |
| Model37 | Front Genet | 32431729 | LGG | Zhang2 |
| Model38 | Front Oncol | 32500034 | Glioma | Lin3 |
| Model39 | J Cell Physiol | 32519365 | LGG | Wang8 |
| Model40 | Front Oncol | 32528873 | GBM | Cai2 |
| Model41 | Oncol Lett | 32952656 | GBM | Chen3 |
| Model42 | Biomed Res Int | 32964017 | Glioma | Fan |
| Model43 | J Cell Physiol | 32964440 | GBM | Chen4 |
| Model44 | Sci Rep | 32968155 | GBM | Chen5 |
| Model45 | Front Oncol | 32974125 | Glioma | Wang9 |
| Model46 | Am J Transl Res | 33042422 | Glioma | Qu1 |
| Model47 | Genomics | 33069830 | LGG | Lin4 |
| Model48 | Aging (Albany NY) | 33186124 | Glioma | Li8 |
| Model49 | Front Oncol | 33194668 | Glioma | Xu1 |
| Model50 | BMC Cancer | 33198677 | Glioma | Li9 |
| Model51 | Sci Rep | 33230136 | Glioma | Feng |
| Model52 | Cancer Med | 33264518 | Glioma | Qu2 |
| Model53 | Front Immunol | 33363544 | LGG | Zhang3 |
| Model54 | Oncol Lett | 33376548 | Glioma | Wang10 |
| Model55 | Front Oncol | 33381460 | LGG | Xiao |
| Model56 | J Cell Mol Med | 33594759 | LGG | Zheng1 |
| Model57 | Front Cell Dev Biol | 33634092 | Glioma | Tu |
| Model58 | Mol Ther Nucleic Acids | 33665000 | LGG | Xu2 |
| Model59 | Front Genet | 33732284 | Glioma | Tan |
| Model60 | Front Oncol | 33768004 | LGG | Guo |
| Model61 | J Oncol | 33790966 | Glioma | Gong |
| Model62 | Medicine (Baltimore) | 33879726 | Glioma | Liu4 |
| Model63 | Biosci Rep | 33969375 | LGG | Lin5 |
| Model64 | J Int Med Res | 34024193 | GBM | Lin6 |
| Model65 | Biomed Res Int | 34212046 | LGG | Zheng2 |

**Supplement Table 5. Basic Characteristics of the Population Characteristics**

| Characteristic | REMBRANDT, N = 475 | TCGA, N = 702 | TIANTAN-301, N = 301 | TIANTAN-325, N = 325 | TIANTAN-693, N = 693 |
| --- | --- | --- | --- | --- | --- |
| Grade |  |  |  |  |  |
| WHO 2 | 92 (27%) | 216 (35%) | 117 (39%) | 103 (32%) | 188 (27%) |
| WHO 3 | 70 (20%) | 241 (40%) | 57 (19%) | 79 (25%) | 255 (37%) |
| WHO 4 | 181 (53%) | 152 (25%) | 124 (42%) | 139 (43%) | 249 (36%) |
| Gender |  |  |  |  |  |
| Female | 121 (37%) | 255 (42%) | 121 (40%) | 122 (38%) | 295 (43%) |
| Male | 203 (63%) | 354 (58%) | 180 (60%) | 203 (62%) | 398 (57%) |
| Age | - | 47 (35, 59) | 42 (34, 52) | 42 (36, 51) | 43 (34, 51) |
| Radio_status |  |  |  |  |  |
| Treated | 0 (NA%) | 0 (NA%) | 237 (84%) | 244 (79%) | 510 (79%) |
| Untreated | 0 (NA%) | 0 (NA%) | 46 (16%) | 66 (21%) | 136 (21%) |
| Chemo status |  |  |  |  |  |
| TMZ treated | 0 (NA%) | 0 (NA%) | 133 (48%) | 193 (63%) | 486 (75%) |
| Untreated | 0 (NA%) | 0 (NA%) | 144 (52%) | 111 (37%) | 161 (25%) |
| IDH mutation |  |  |  |  |  |
| Mutant | 0 (NA%) | 428 (65%) | 134 (45%) | 175 (54%) | 356 (55%) |
| Wildtype | 0 (NA%) | 234 (35%) | 165 (55%) | 149 (46%) | 286 (45%) |
| Unknown | 475 | 40 | 2 | 1 | 51 |
| 1p19q Codeletion status |  |  |  |  |  |
| Codel | 24 (14%) | 169 (25%) | 16 (17%) | 67 (21%) | 145 (23%) |
| Non-codel | 150 (86%) | 495 (75%) | 76 (83%) | 250 (79%) | 478 (77%) |
| MGMTp methylation status |  |  |  |  |  |
| methylated | 0 (NA%) | 0 (NA%) | 99 (35%) | 157 (51%) | 315 (58%) |
| un-methylated | 0 (NA%) | 0 (NA%) | 187 (65%) | 149 (49%) | 227 (42%) |

^1^n / N (%); ^2^Mean (SD); Chinese Glioma Genome Atlas (CGGA); The Cancer Genome Atlas (TCGA).
